# Supplementary material for: CD70 drives cSCC growth by linking DNA damage response, inflammation, and tumor–stromal signaling
Source: Cell Death Dis. 2026 Jun 11;17(1):736. doi: 10.1038/s41419-026-08876-x (PMC13478633; doi:10.1038/s41419-026-08876-x)
Supplement: Supplementary file 1 [file 41419_2026_8876_MOESM10_ESM.pdf]

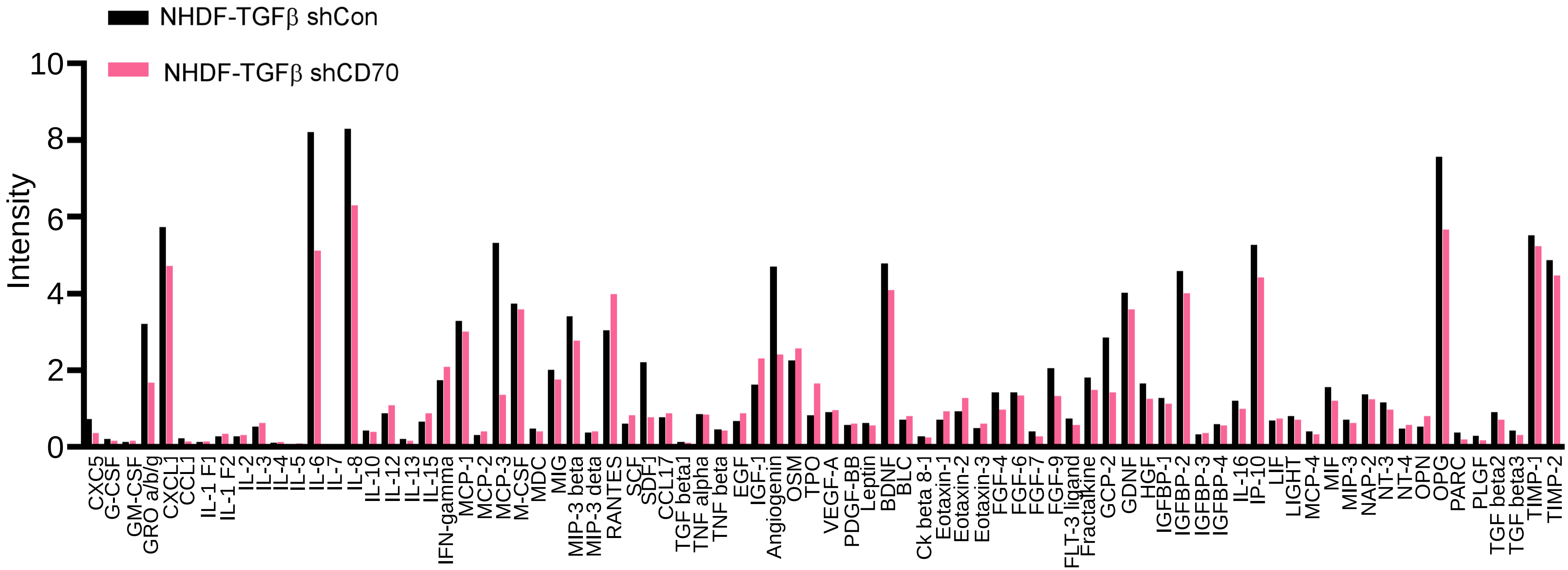

NHDF-TGFβ shCon
NHDF-TGFβ shCD70
Intensity
0
2
4
6
8
10
CXC5
G-CSF
GM-CSF
GRO a/b/g
CXCL1
CCL1
IL-1 F1
IL-1 F2
IL-2
IL-3
IL-4
IL-5
IL-6
IL-7
IL-8
IL-10
IL-12
IL-13
IL-15
IFN-gamma
MCP-1
MCP-2
MCP-3
M-CSF
MDC
MIG
MIP-3 beta
MIP-3 deta
RANTES
SCF
SDF1
CCL17
TGF beta1
TNF alpha
TNF beta
EGF
IGF-1
Angiogenin
OSM
TPO
VEGF-A
PDGF-BB
Leptin
BDNF
BLC
Ck beta 8-1
Eotaxin-1
Eotaxin-2
Eotaxin-3
FGF-4
FGF-6
FGF-7
FGF-9
FLT-3 ligand
Fractalkine
GCP-2
GDNF
HGF
IGFBP-1
IGFBP-2
IGFBP-3
IGFBP-4
IL-16
IP-10
LIF
LIGHT
MCP-4
MIF
MIP-3
NAP-2
NT-3
NT-4
OPN
OPG
PARC
PLGF
TGF beta2
TGF beta3
TIMP-1
TIMP-2
