# Supplementary material for: CD70 drives cSCC growth by linking DNA damage response, inflammation, and tumor–stromal signaling
Source: Cell Death Dis. 2026 Jun 11;17(1):736. doi: 10.1038/s41419-026-08876-x (PMC13478633; doi:10.1038/s41419-026-08876-x)
Supplement: Supplementary file 2 [file 41419_2026_8876_MOESM11_ESM.pdf]

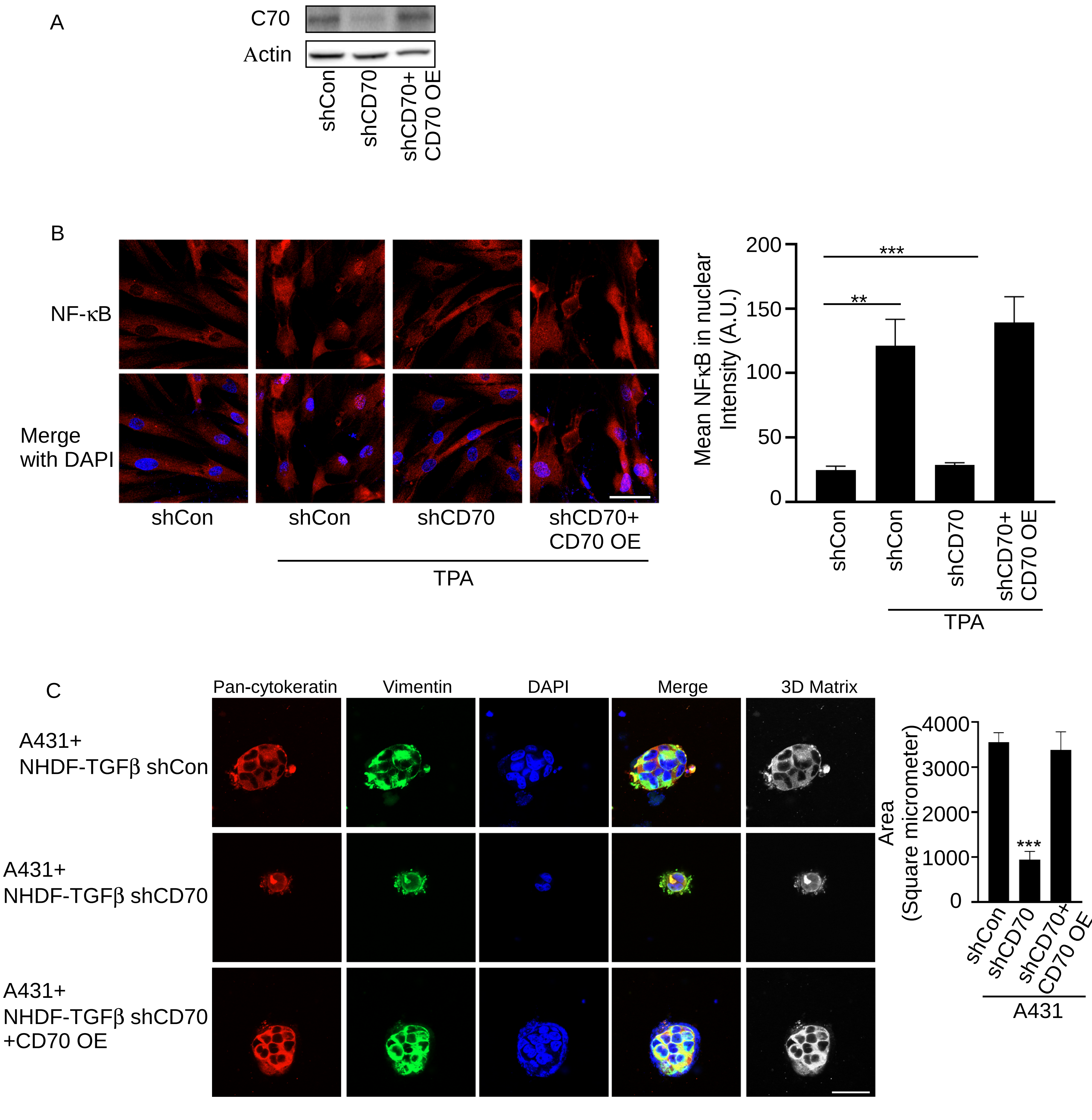

A
C70
Actin
shCon
shCD70
shCD70+
CD70 OE
B
NF-κB
Merge
with DAPI
shCon
shCon
shCD70
shCD70+
CD70 OE
TPA
Mean NFκB in nuclear
Intensity (A.U.)
200
150
100
50
0
***
**
shCon
shCon
shCD70
shCD70+
CD70 OE
TPA
C
Pan-cytokeratin
Vimentin
DAPI
Merge
3D Matrix
A431+
NHDF-TGFβ shCon
A431+
NHDF-TGFβ shCD70
A431+
NHDF-TGFβ shCD70
+CD70 OE
Area
(Square micrometer)
4000
3000
2000
1000
0
***
shCon
shCD70
shCD70+
CD70 OE
A431
