# Supplementary material for: CD70 drives cSCC growth by linking DNA damage response, inflammation, and tumor–stromal signaling
Source: Cell Death Dis. 2026 Jun 11;17(1):736. doi: 10.1038/s41419-026-08876-x (PMC13478633; doi:10.1038/s41419-026-08876-x)

Fig2A

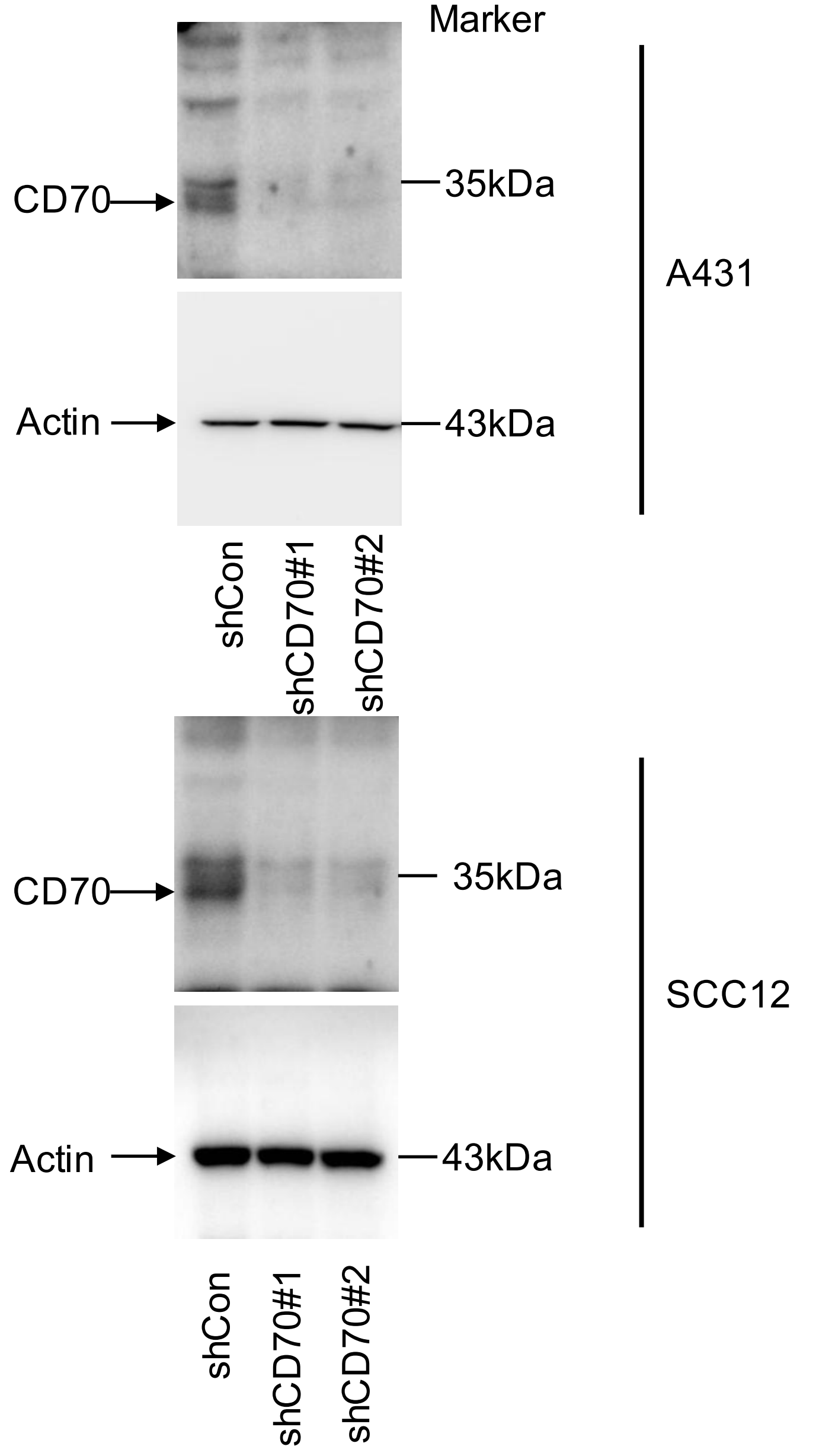
Marker
CD70
35kDa
Actin
43kDa
A431
shCon
shCD70#1
shCD70#2
CD70
35kDa
Actin
43kDa
SCC12
shCon
shCD70#1
shCD70#2

Fig3A

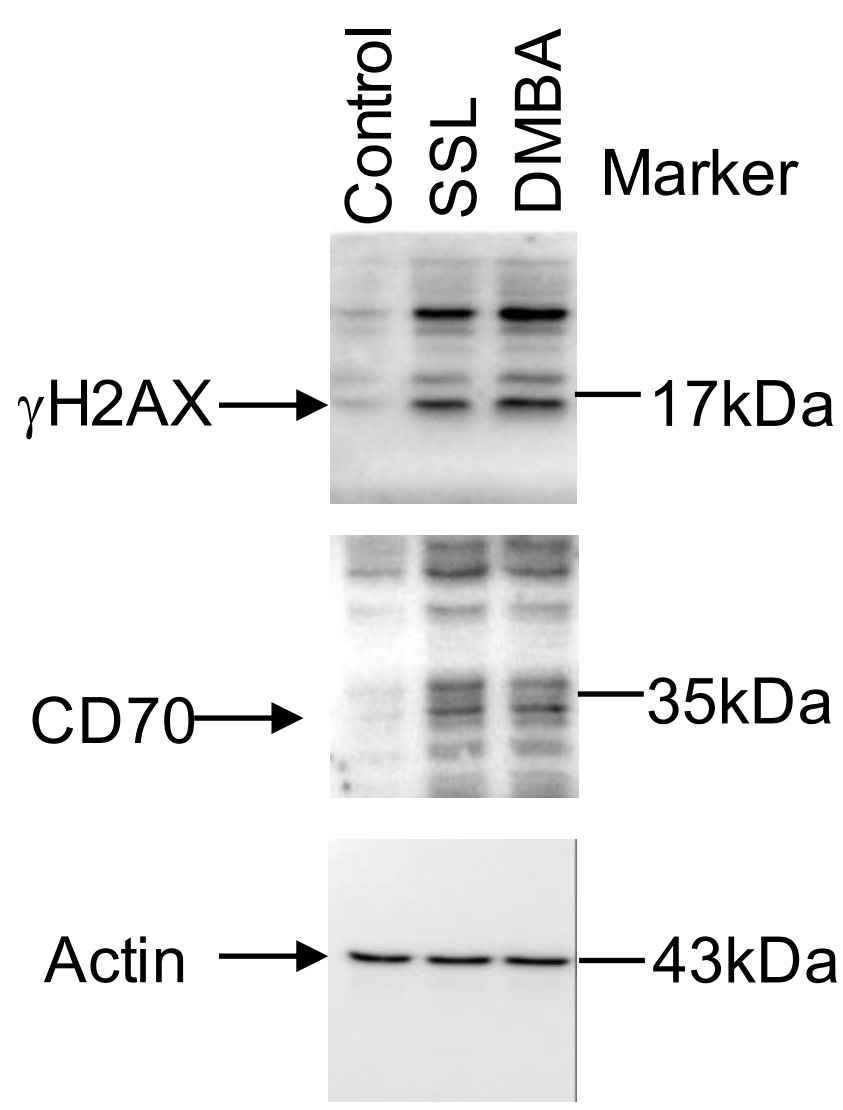

Fig3D

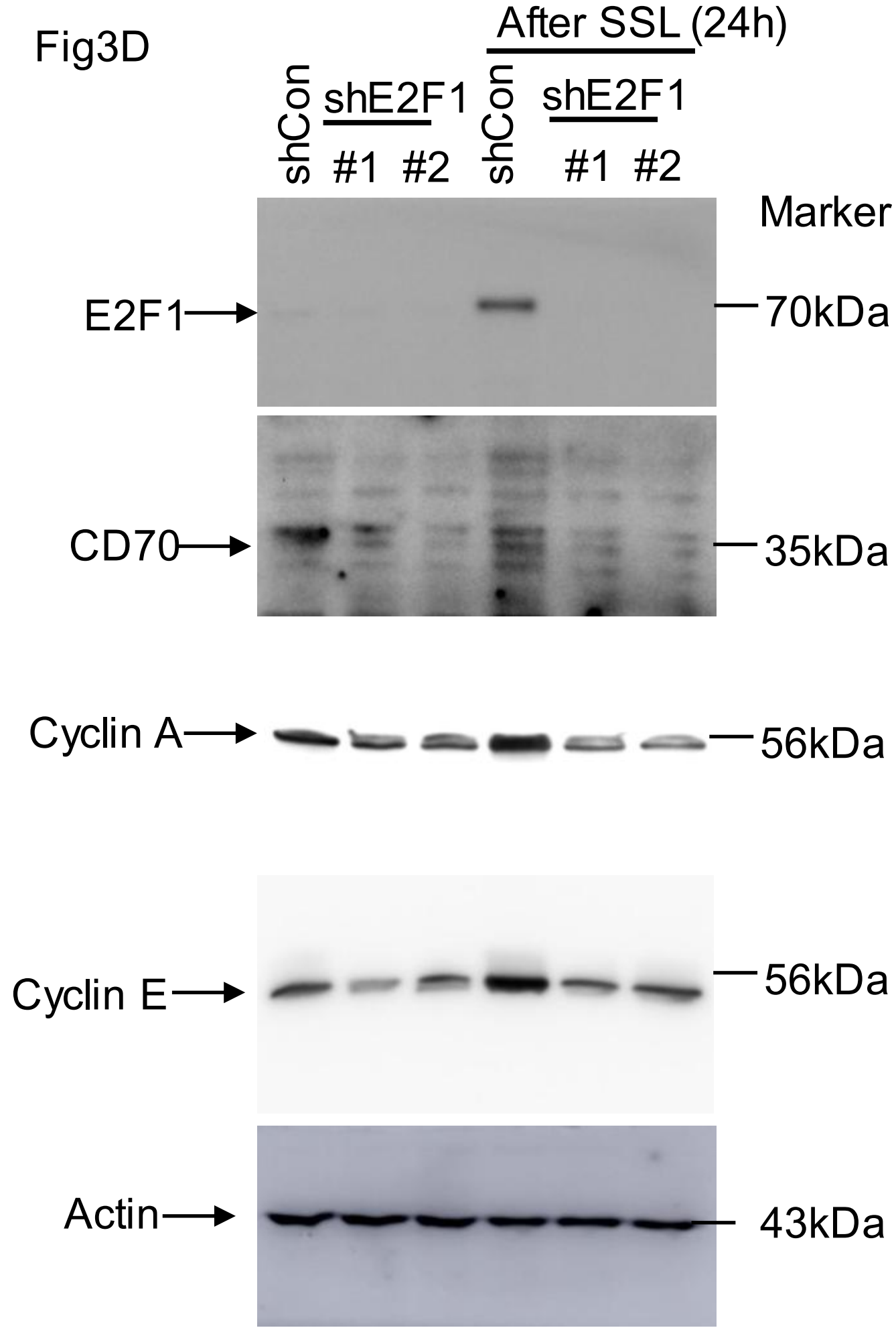

Fig3F

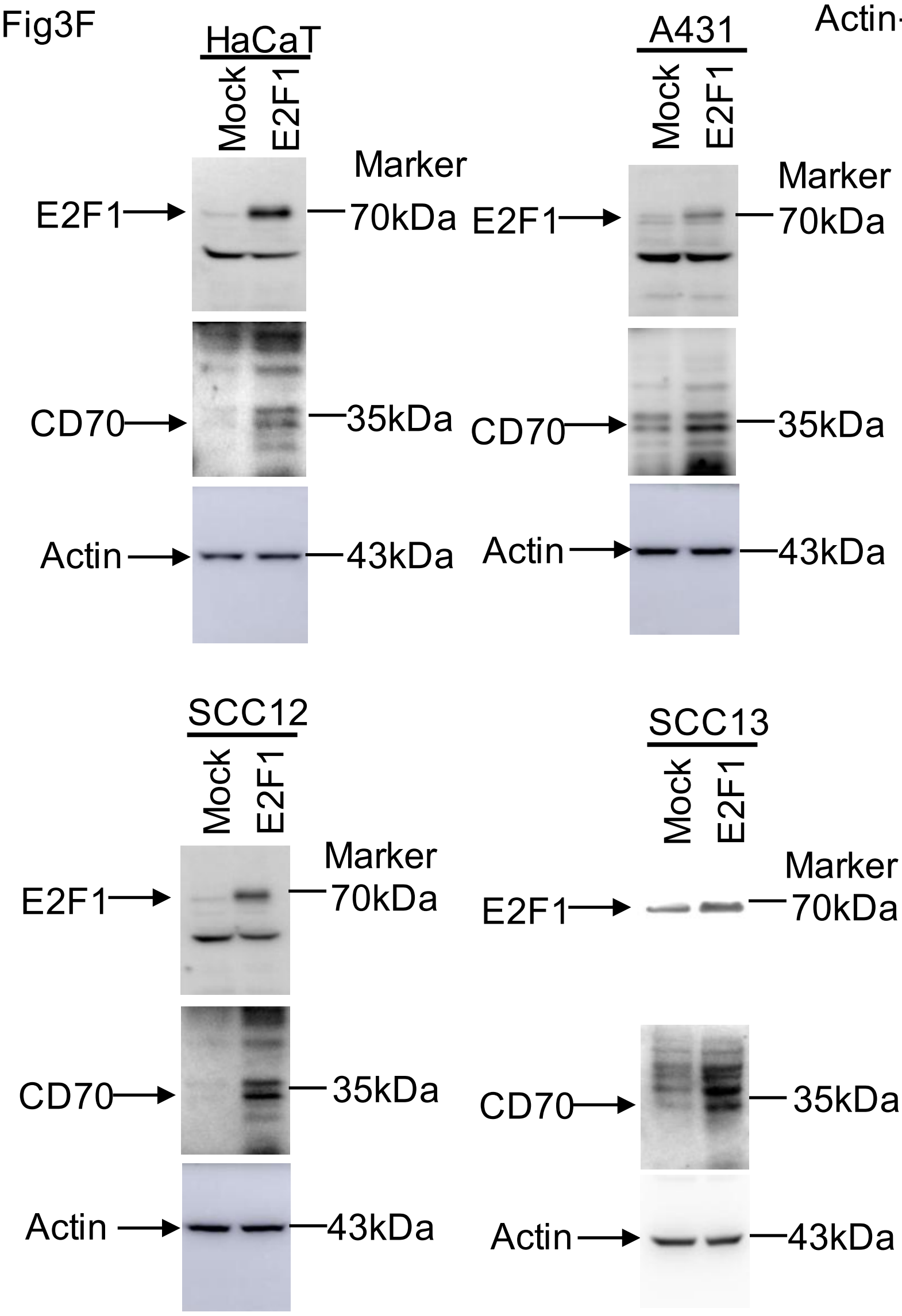

Fig3N

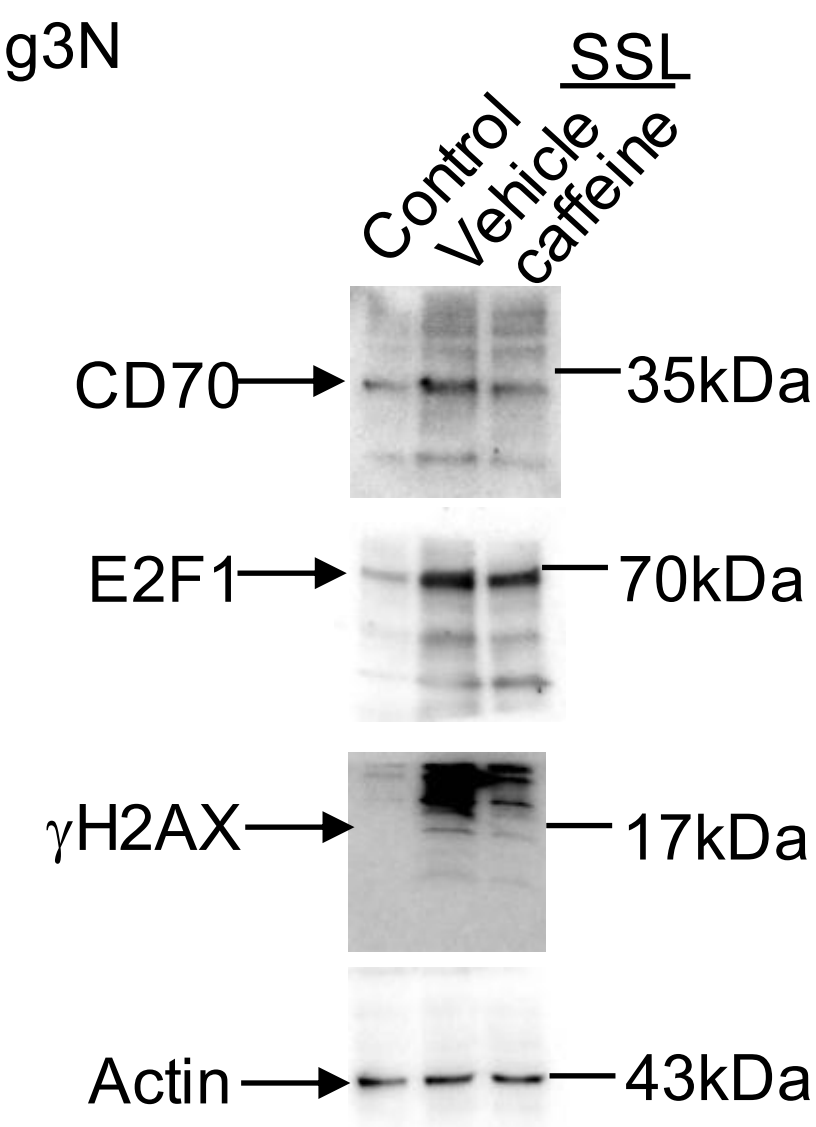

Fig4D

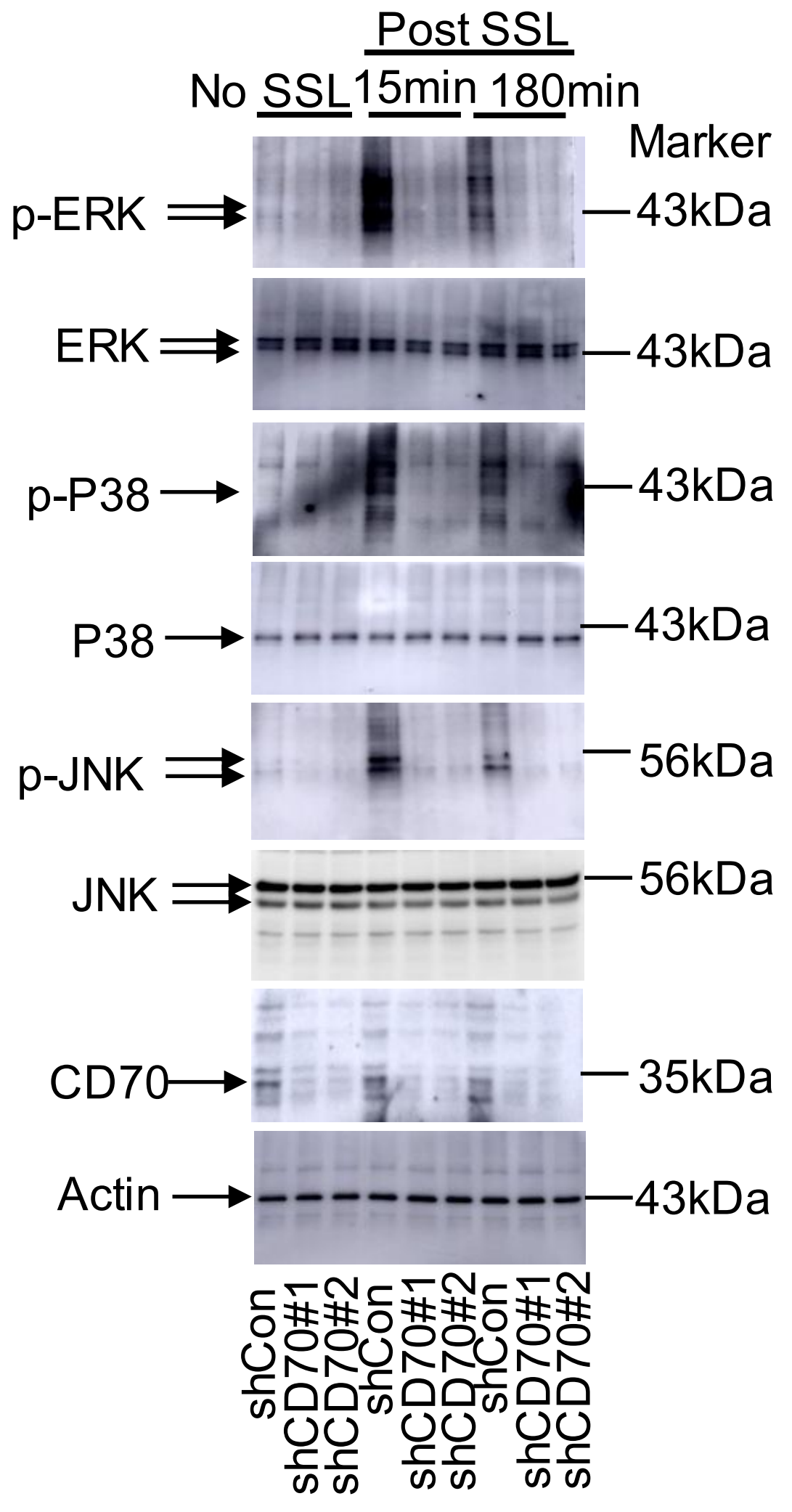
Post SSL
No SSL
15min
180min
Marker
p-ERK
43kDa
ERK
43kDa
p-P38
43kDa
P38
43kDa
p-JNK
56kDa
JNK
56kDa
CD70
35kDa
Actin
43kDa
shCon
shCD70#1
shCD70#2
shCon
shCD70#1
shCD70#2
shCon
shCD70#1
shCD70#2

Fig4G

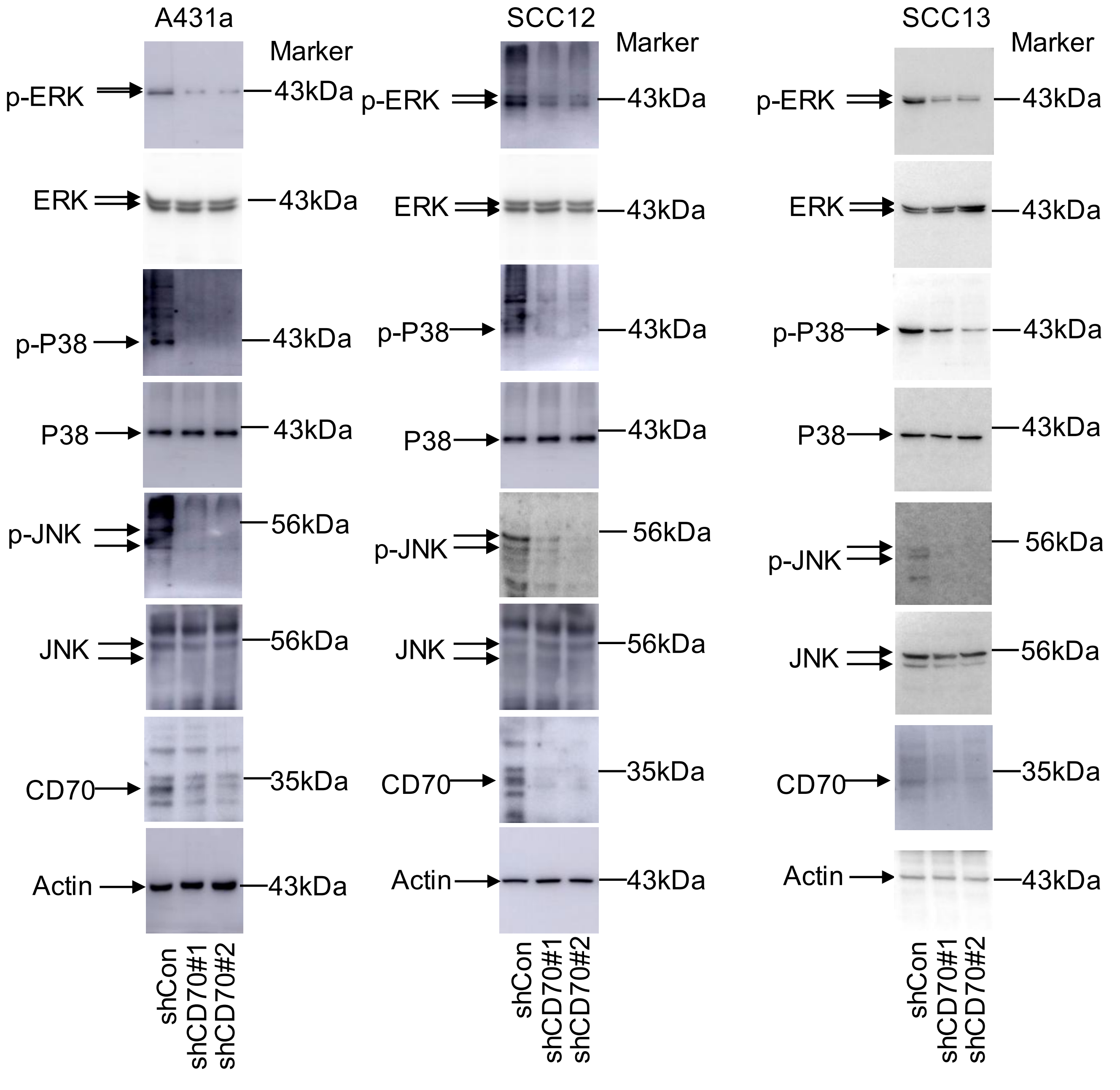

A431a
SCC12
SCC13
Marker
p-ERK
ERK
p-P38
P38
p-JNK
JNK
CD70
Actin
43kDa
56kDa
35kDa
shCon
shCD70#1
shCD70#2

Fig4J

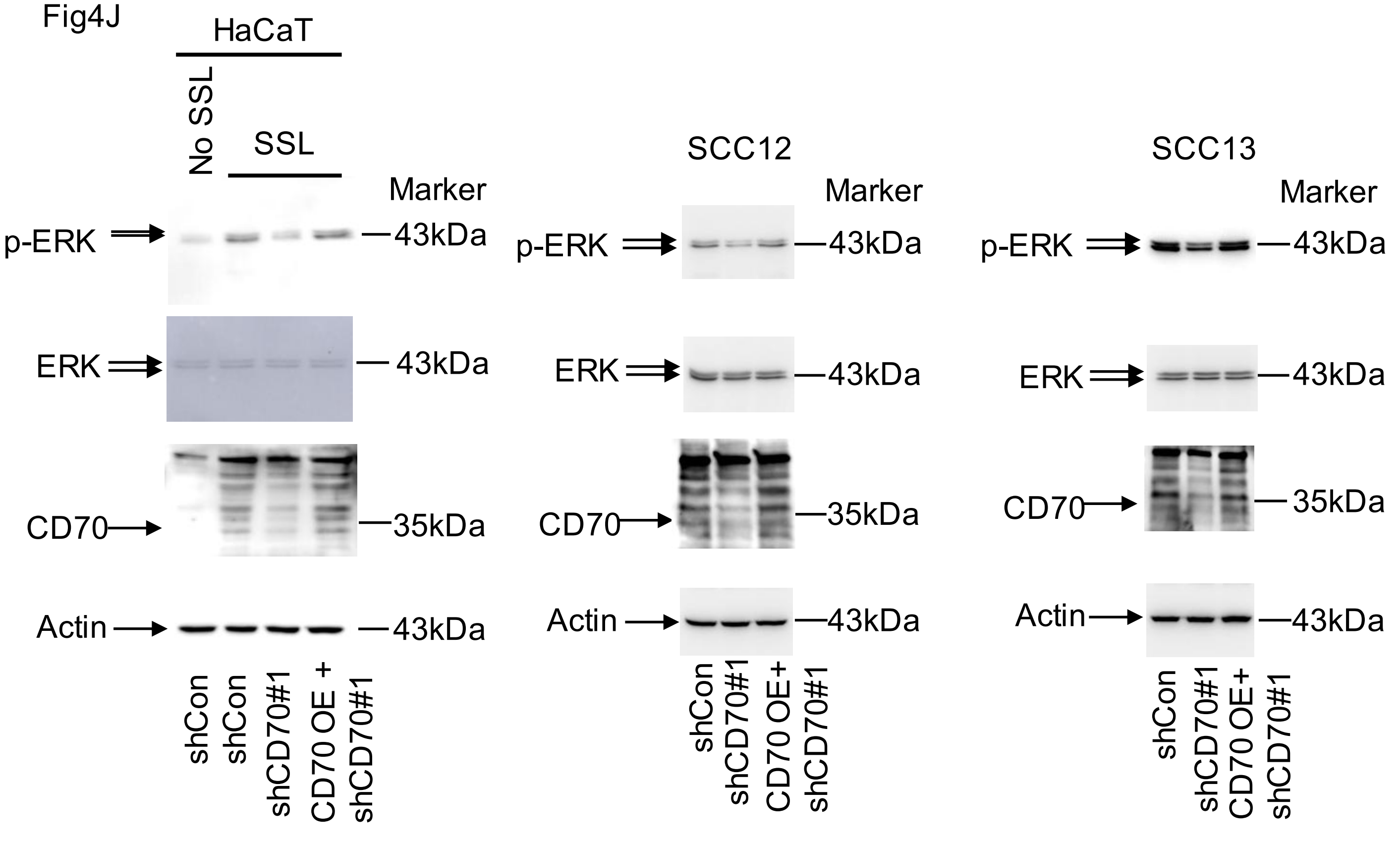
HaCaT
No SSL
SSL
Marker
p-ERK
43kDa
ERK
43kDa
CD70
35kDa
Actin
43kDa
shCon
shCon
shCD70#1
CD70 OE + shCD70#1
SCC12
Marker
p-ERK
43kDa
ERK
43kDa
CD70
35kDa
Actin
43kDa
shCon
shCD70#1
CD70 OE+ shCD70#1
SCC13
Marker
p-ERK
43kDa
ERK
43kDa
CD70
35kDa
Actin
43kDa
shCon
shCD70#1
CD70 OE+ shCD70#1

Fig5A

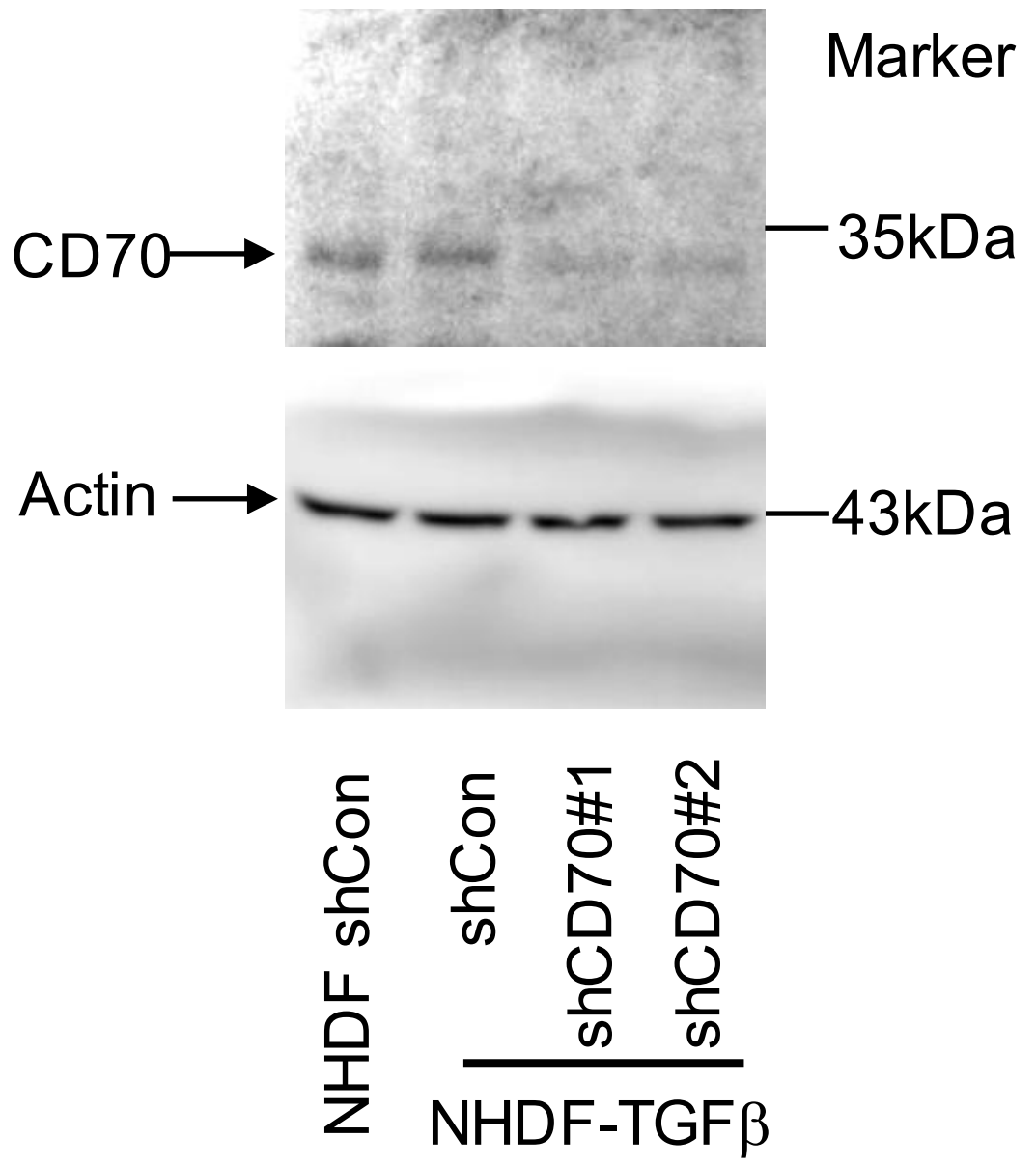
Marker
CD70
35kDa
Actin
43kDa
NHDF shCon
shCon
shCD70#1
shCD70#2
NHDF-TGFβ

Fig5F

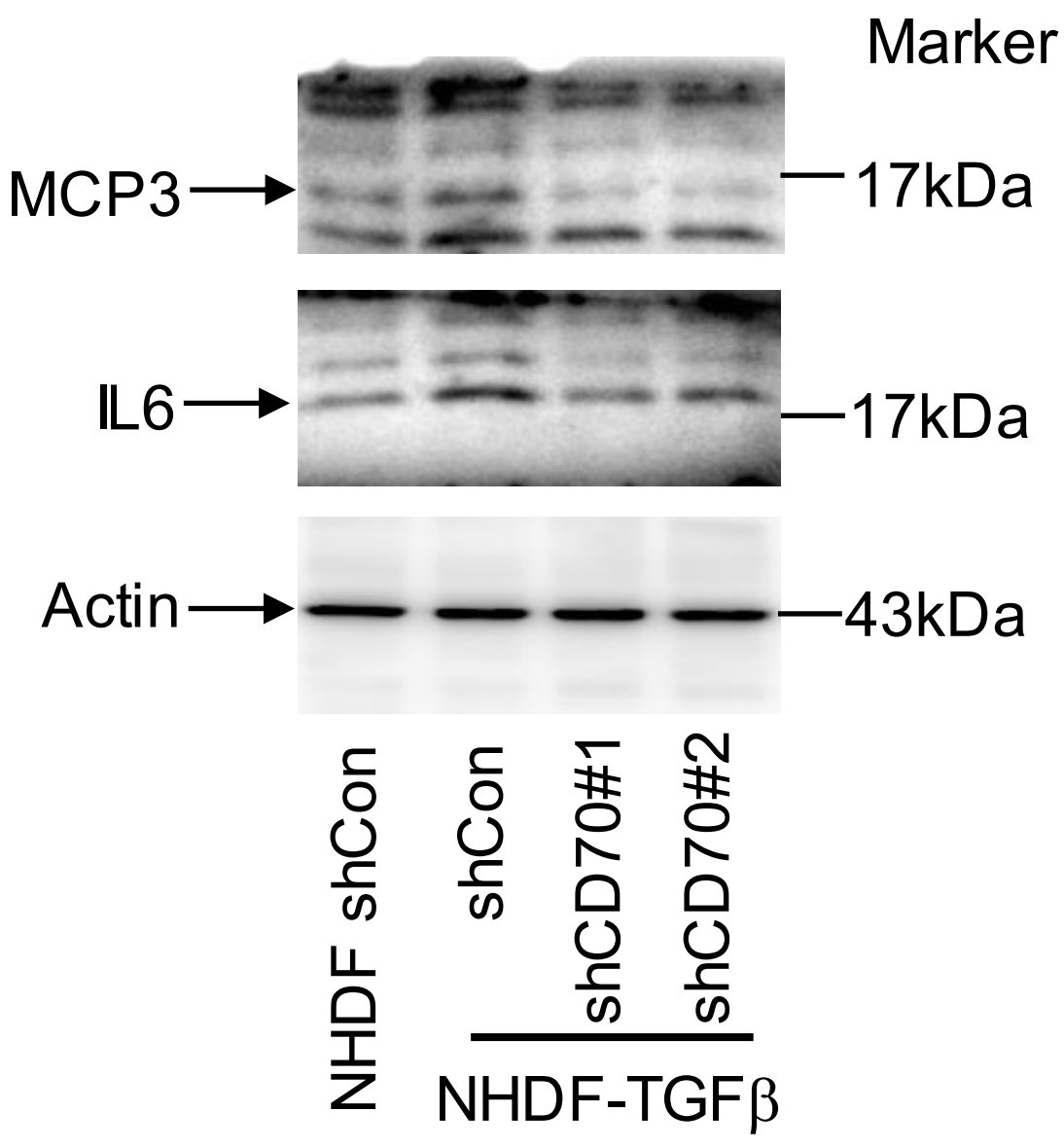
Marker
MCP3
17kDa
IL6
17kDa
Actin
43kDa
NHDF shCon
shCon
shCD70#1
shCD70#2
NHDF-TGFβ

Fig S2A

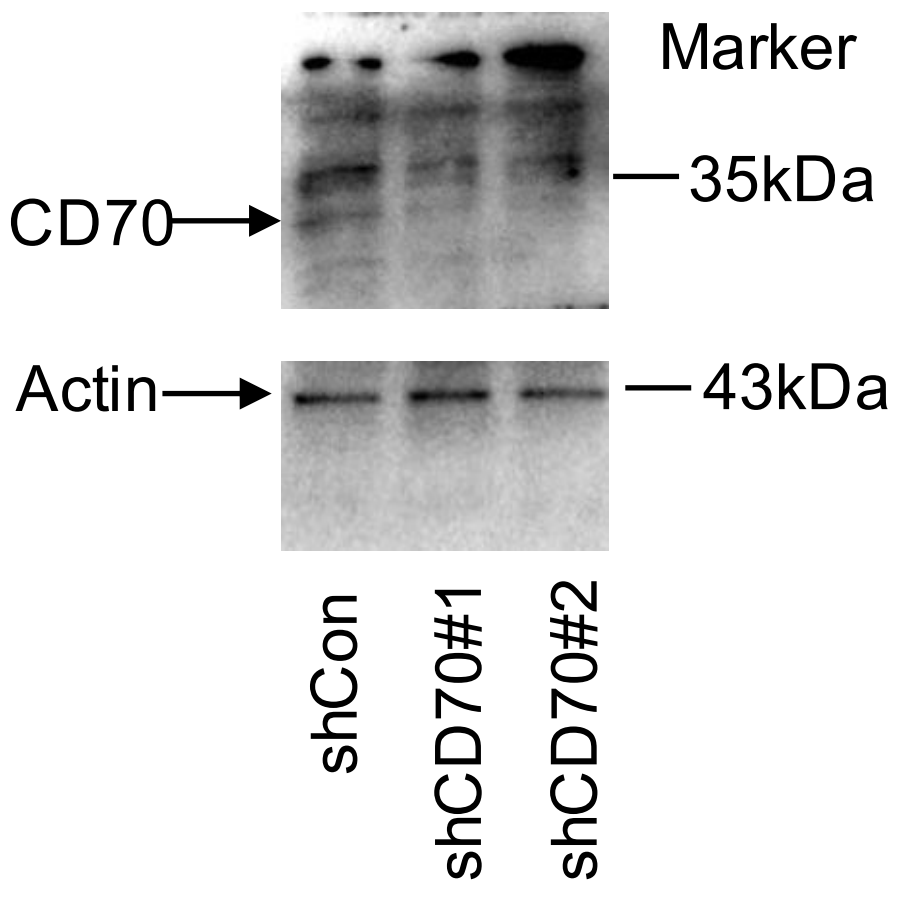

Fig S3A

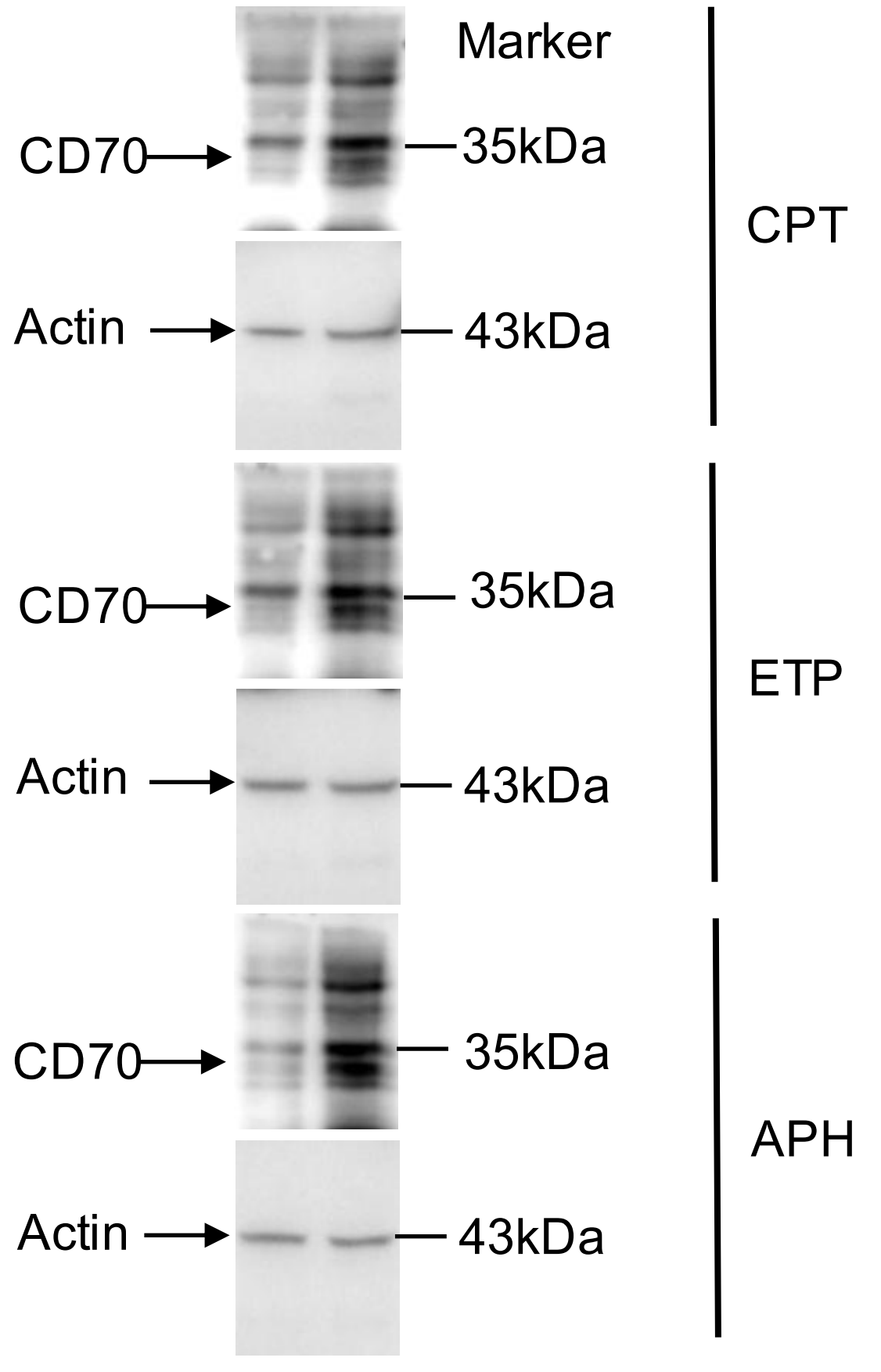

Fig S3C

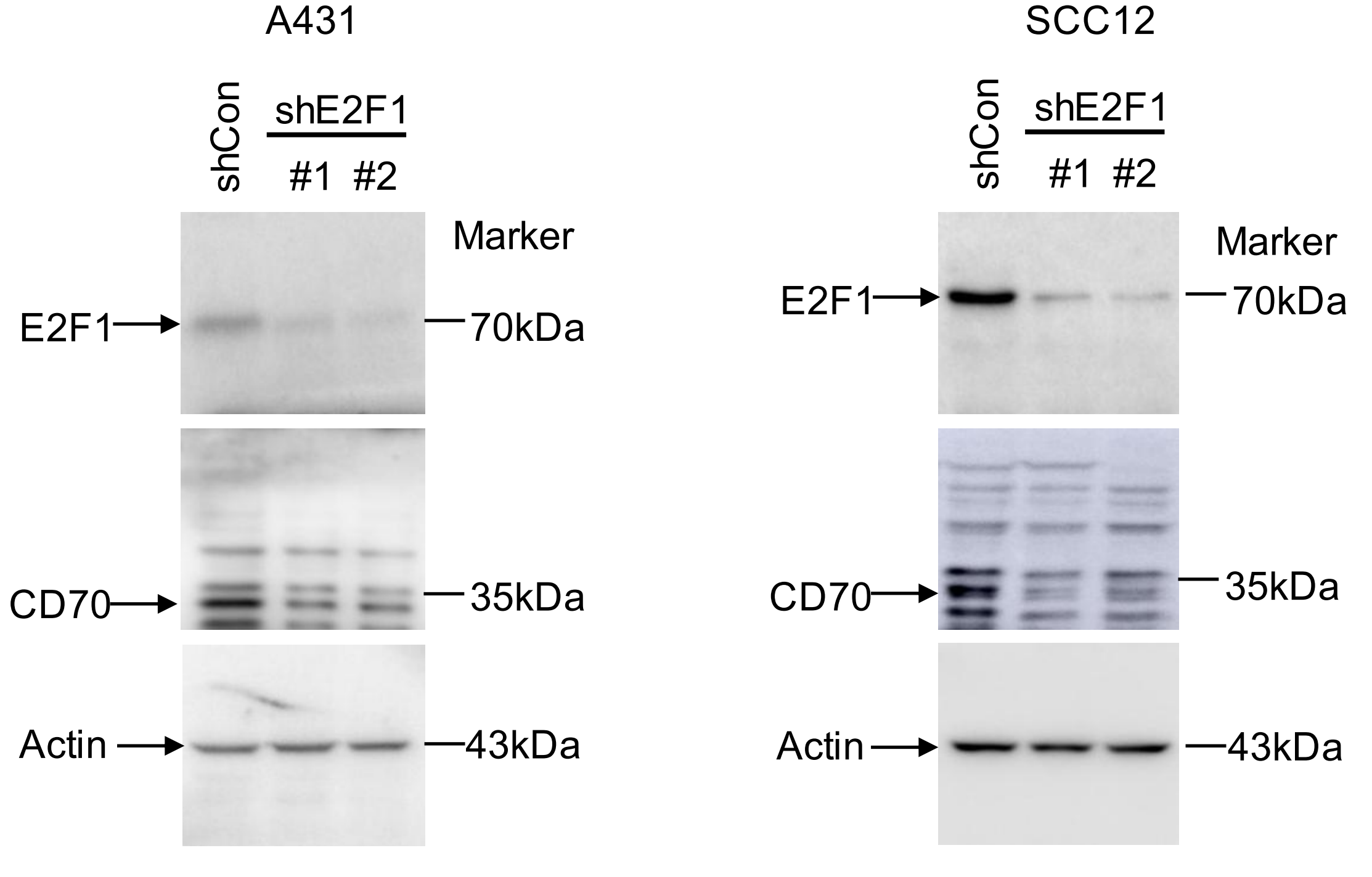

Fig S4B

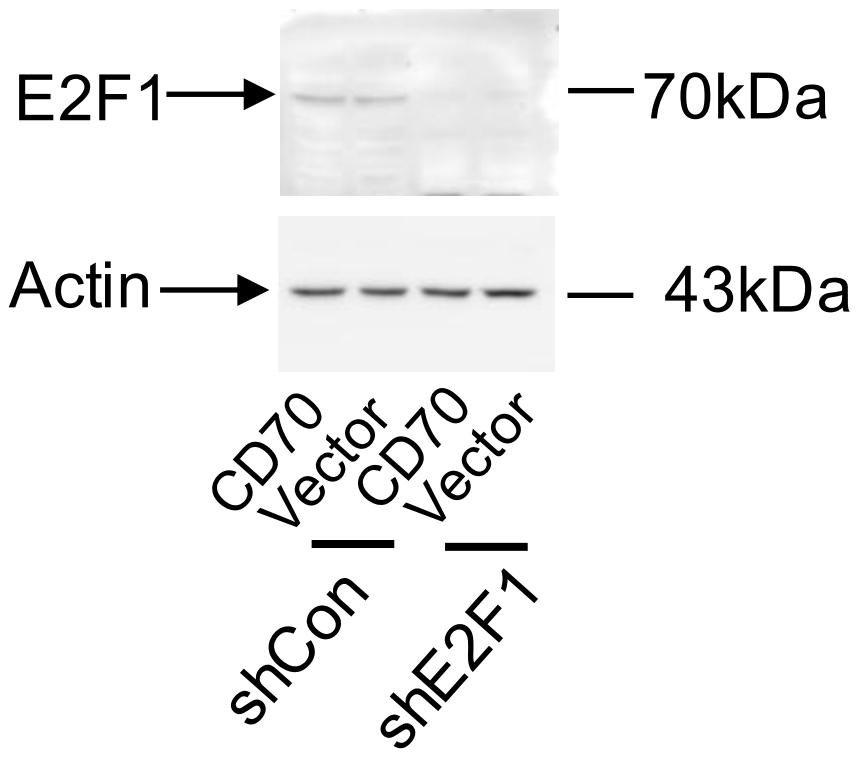

Fig S4C

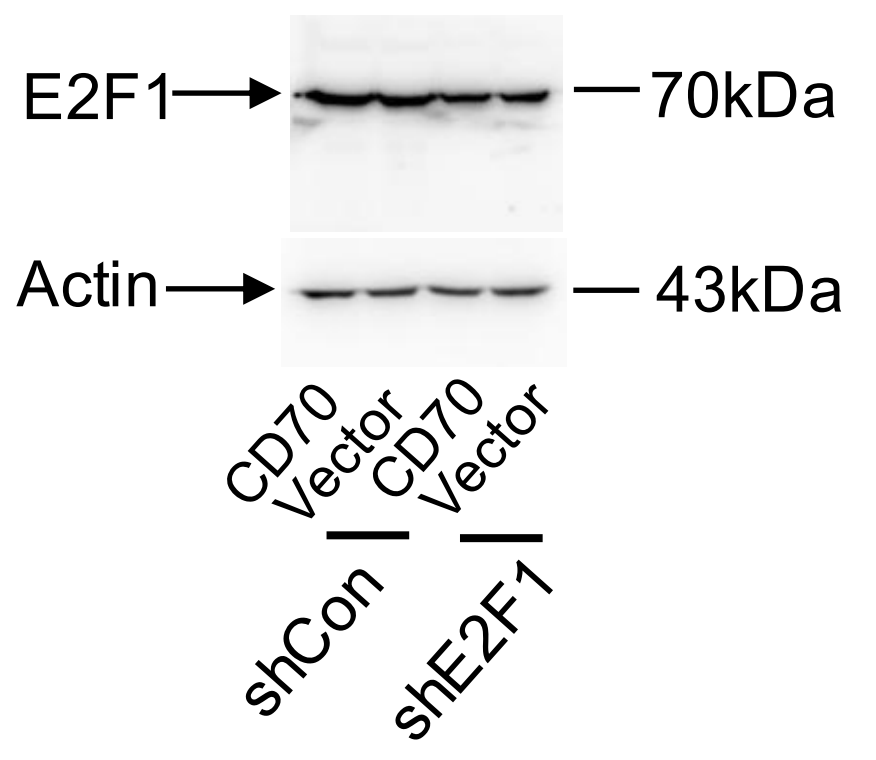

Fig S5A

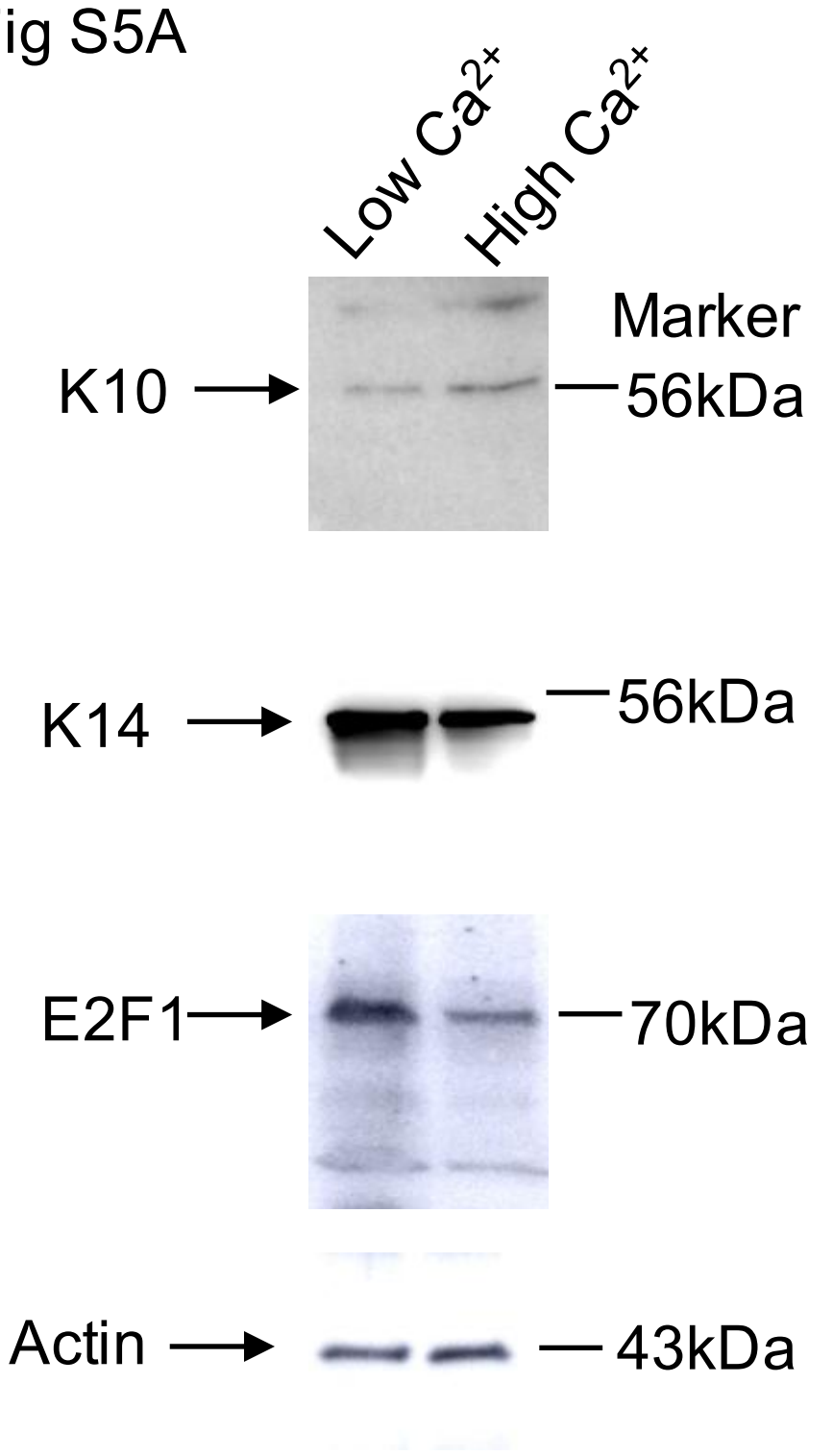

Fig S5B

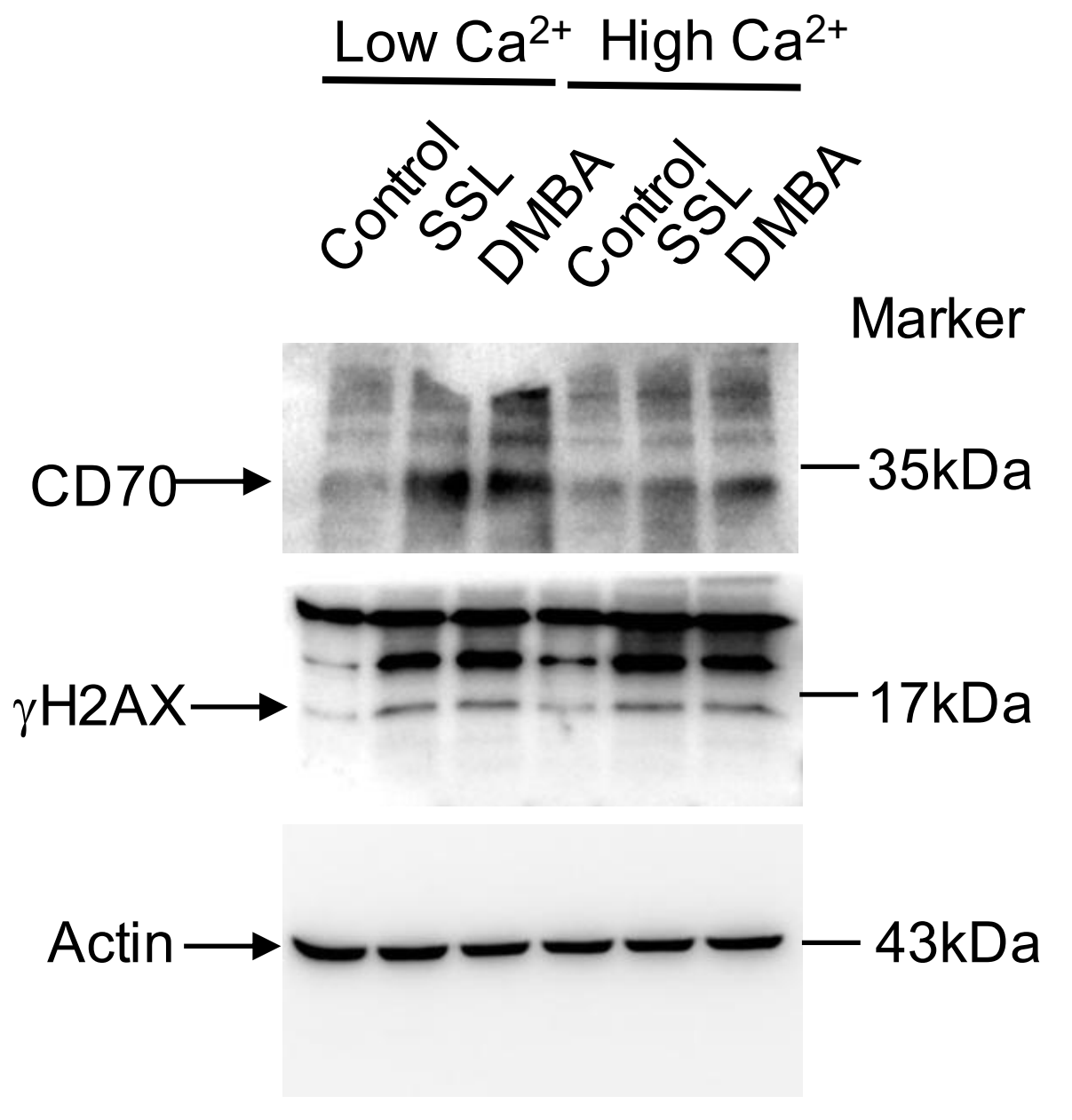

Fig S5C

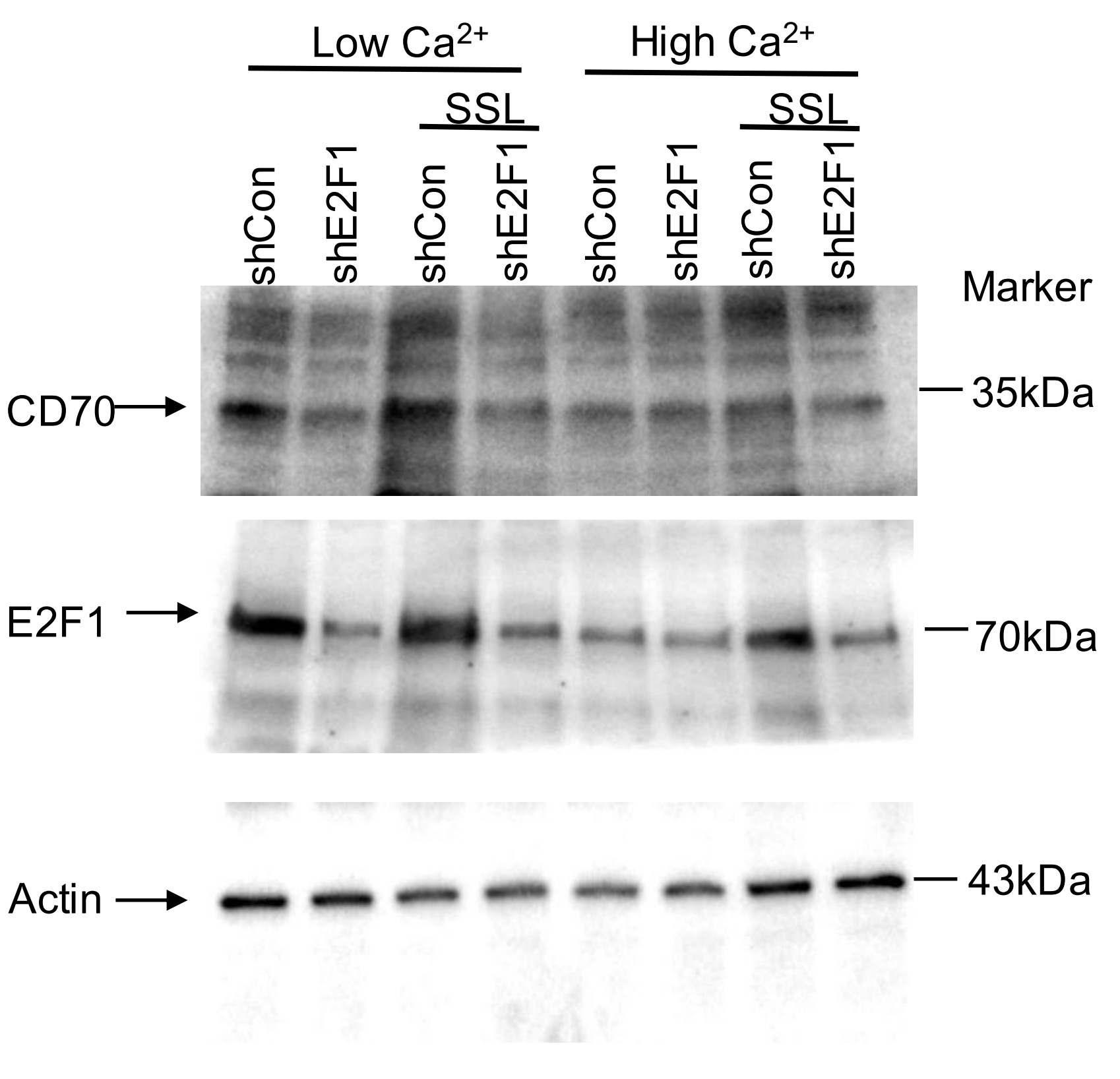

Fig S6A

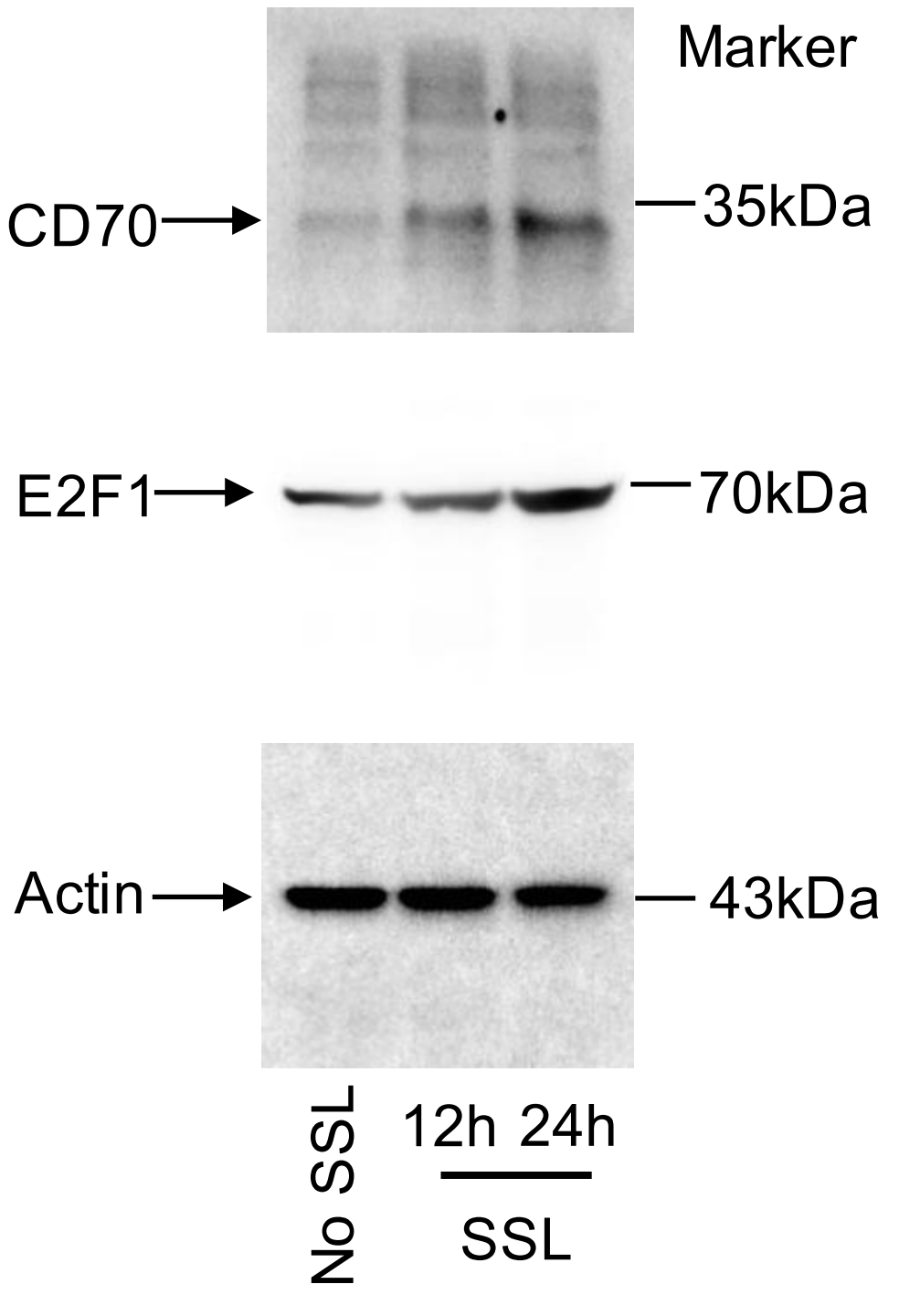

Fig S11A

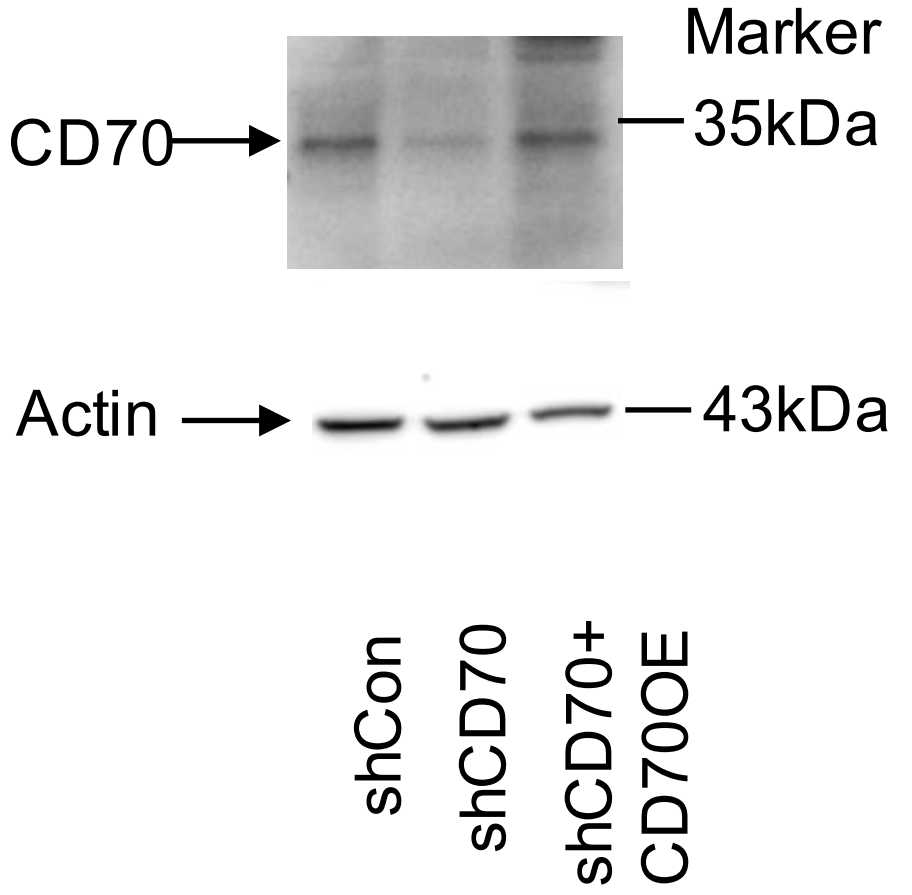

Supplement: Supplementary file 3 [file 41419_2026_8876_MOESM14_ESM.pdf]
