# Supplementary material for: CD70 drives cSCC growth by linking DNA damage response, inflammation, and tumor–stromal signaling
Source: Cell Death Dis. 2026 Jun 11;17(1):736. doi: 10.1038/s41419-026-08876-x (PMC13478633; doi:10.1038/s41419-026-08876-x)
Supplement: Supplementary file 4 [file 41419_2026_8876_MOESM15_ESM.pdf]

SPRINGER NATURE

Corresponding Author Name: Tianshun Zhan(
Manuscript Number: CDDIS-25-740(

Reporting Summary

Springer Nature wishes to improve the reproducibility of the work that we publish. This checklist is used to ensure good reporting standards and to improve the reproducibility. Please respond completely to all questions relevant to your manuscript. For more information, please read the journal's Guide to Authors.

☑ **Check here to confirm that the following information is available in the Material & Methods section:**

- **The exact sample size (*n*)** for each experimental group/condition, given as a number, not a range
- **A description of the sample collection** allowing the reader to understand whether the samples represent technical or biological replicates (including how many animals, litters, culture, etc.)
- **A statement of how many times the experiment shown was replicated in the laboratory**
- **Definitions of statistical methods and measures**: For small sample sizes (n<5) descriptive statistics are not appropriate, instead plot individual data points
    - Very common tests, such as *t*-test, simple χ^2 tests, Wilcoxon and Mann-Whitney tests, can be unambiguously identified by name only, but more complex techniques should be described in the methods section
    - Are tests one-sided or two-sided?
    - Are there adjustments for multiple comparisons?
    - **Statistical test results**, e.g., ***P* values**
    - Definition of **'center values'** as **median or mean**;
    - Definition of **error bars as s.d. or s.e.m. or c.i.**

Please ensure that the answers to the following questions are reported in the manuscript itself. We encourage you to include a specific subsection in the methods section for statistics, reagents and animal models. Below, provide the page number or section and paragraph number.

Statistics and general methods

	**Reported in section/paragraph or page #**
1. How was the sample size chosen to ensure adequate power to detect a pre-specified effect size? (Give section/paragraph or page #)	Sample sizes were not determined by formal statistical power analysis. Instead, sample sizes were selected on the basis of prior experience, preliminary experiments, and
For animal studies, include a statement about sample size estimate even if no statistical methods were used.	For animal experiments, group sizes were chosen to allow detection of biologically meaningful differences while minimizing animal use (Page 11).
2. Describe inclusion/exclusion criteria if samples or animals were excluded from the analysis. Were the criteria pre-established? (Give section/paragraph or page #)	No data were excluded from the analyses (Page 13).
3. If a method of randomization was used to determine how samples/animals were allocated to experimental groups and processed, describe it. (Give section/paragraph or page #)	Mice were randomly assigned to experimental groups(Page 11).
For animal studies, include a statement about randomization even if no randomization was used.	Mice were randomly assigned to experimental groups (Page 11).

4. If the investigator was blinded to the group allocation during the experiment and/or when assessing the outcome, state the extent of blinding. (Give section/paragraph or page #)	Investigators were not blinded to group allocation. However, data collection and analysis were performed using objective and standardized methods to minimize potential bias (Page 13).
For animal studies, include a statement about blinding even if no blinding was done.	Investigators were not blinded to group allocation during animal experiments. However, tumor measurements and
5. For every figure, are statistical tests justified as appropriate?	Yes. Statistical tests were selected based on the distribution and characteristics of the data. Parametric
Do the data meet the assumptions of the tests (e.g., normal distribution)?	Yes. Data were assessed for normality, and appropriate statistical tests were applied accordingly. For RPPA data,
Is there an estimate of variation within each group of data?	Yes. Data are presented as mean ± standard deviation (SD) or standard error (SE), as indicated in the figure legends.
Is the variance similar between the groups that are being statistically compared? (Give section/paragraph or page #)	Variance between groups was assessed and found to be comparable. Statistical tests used are robust to moderate deviations from equal variance (Statistical analysis section).

Reagents

Reported in section/paragraph or page #

6. Report the source of antibodies (vendor and catalog number)	Antibodies are described in the Supplementary Materials and Methods section, including vendor and catalog
7. Identify the source of cell lines and report if they were recently authenticated (e.g., by STR profiling) and tested for mycoplasma contamination	HaCaT and HEK 293T cells were from ATCC (Manassas, VA); A431 and SCC-12 cells from Thermo Fisher Scientific (Waltham, MA); NHDF from Lonza (Walkersville, MD). Primary human keratinocytes derived from neonatal

Animal Models

Reported in section/paragraph or page #

8. Report species, strain, sex and age of animals	Athymic nude mice (Mus musculus), six weeks old,
9. For experiments involving live vertebrates, include a statement of compliance with ethical regulations and identify the committee(s) approving the experiments.	All animal experiments were conducted in accordance with institutional guidelines and were approved by the University of Minnesota Institutional Animal Care and Use Committee (IACUC, Protocol ID 2404-42009A).

10. We recommend consulting the ARRIVE guidelines (*PLoS Biol.* **8**(6), e1000412,2010) to ensure that other relevant aspects of animal studies are adequately reported.

Human subjects

	Reported in section/paragraph or page #
11. Identify the committee(s) approving the study protocol.	Human samples were collected under an Institutional Review Board (IRB), approved protocol at the University of Arizona
12. Include a statement confirming that informed consent was obtained from all subjects.	Informed consent was obtained from all subjects prior to sample collection.
13. For publication of patient photos, include a statement confirming that consent to publish was obtained.	n/a
14. Report the clinical trial registration number (at ClinicalTrials.gov or equivalent).	n/a

15. For phase II and III randomized controlled trials, please refer to the CONSORT statement and submit the CONSORT checklist with your submission.

16. For tumor marker prognostic studies, we recommend that you follow the REMARK reporting guidelines.

Data deposition

	Reported in section/paragraph or page #
17. Provide accession codes for deposited data. Data deposition in a public repository is mandatory for:<br>a. Protein, DNA and RNA sequences<br>b. Macromolecular structures<br>c. Crystallographic data for small molecules<br>d. Microarray data	The RNA-seq data generated in this study will be deposited in a public database (e.g., GEO) and will be made available upon publication. In the meantime, data are available from the corresponding author upon reasonable request.

Deposition is strongly recommended for many other datasets for which structured public repositories exist; more details on our data policy are available in the Guide to Authors. We encourage the provision of other source data in supplementary information or in unstructured repositories such as Figshare and Dryad. We encourage publication of Data Descriptors (see Scientific Data) to maximize data reuse.

18. If computer code was used to generate results that are central to the paper's conclusions, include a statement in the Methods section under "**Code availability**" to indicate whether and how the code can be accessed. Include version information as necessary and any restrictions on availability.	No custom code was used that is central to the conclusions of this study. Standard software (e.g., GraphPad Prism and R) was used for analysis.
