# Supplementary material for: CD70 drives cSCC growth by linking DNA damage response, inflammation, and tumor–stromal signaling
Source: Cell Death Dis. 2026 Jun 11;17(1):736. doi: 10.1038/s41419-026-08876-x (PMC13478633; doi:10.1038/s41419-026-08876-x)
Supplement: Supplementary file 5 [file 41419_2026_8876_MOESM1_ESM.pdf]

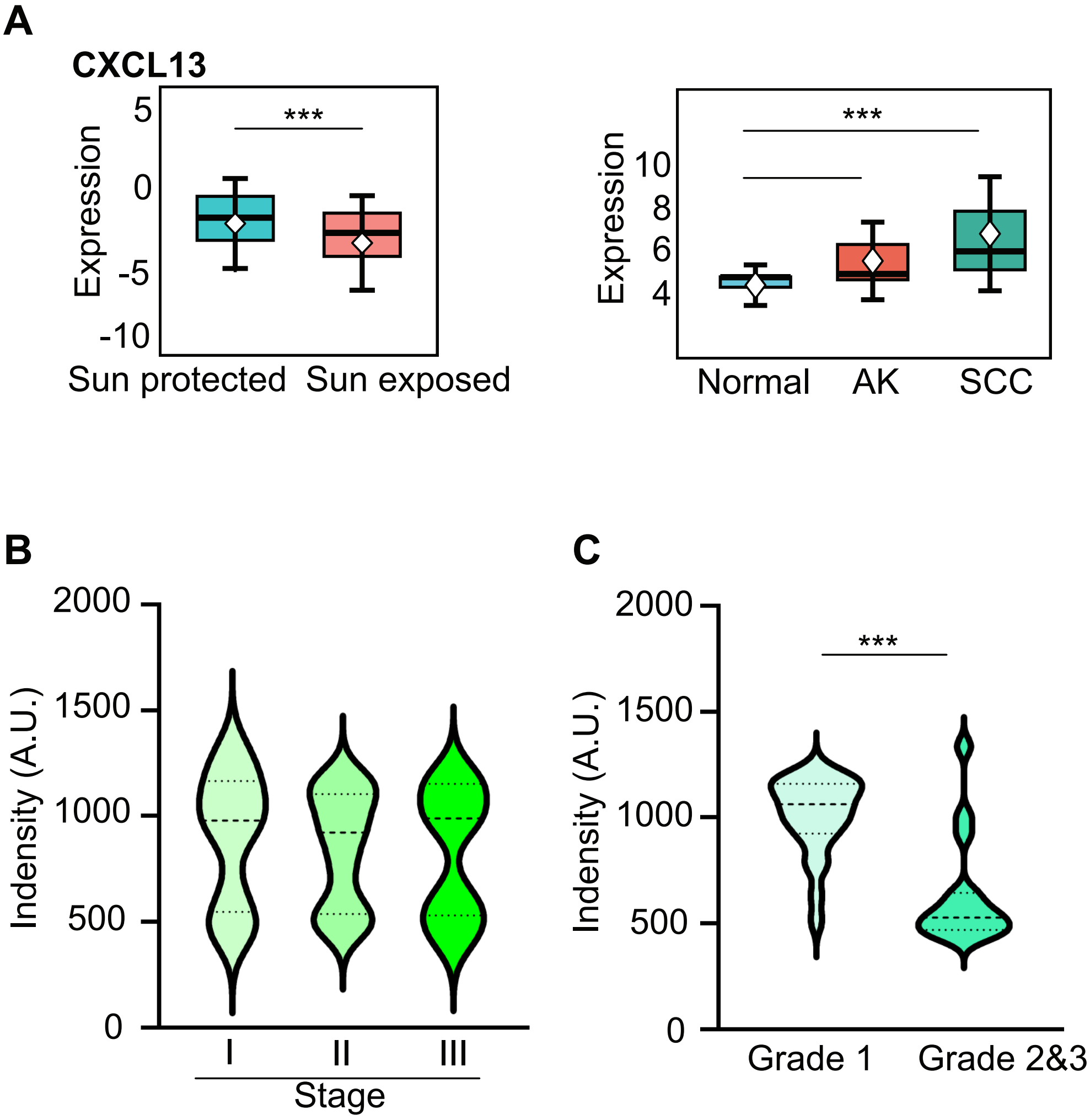

A
CXCL13
Expression
5
0
-5
-10
***
Sun protected
Sun exposed
Expression
10
8
6
4
***
Normal
AK
SCC
B
Indensity (A.U.)
2000
1500
1000
500
0
I
II
III
Stage
C
Indensity (A.U.)
2000
1500
1000
500
0
***
Grade 1
Grade 2&3
