# Supplementary material for: CD70 drives cSCC growth by linking DNA damage response, inflammation, and tumor–stromal signaling
Source: Cell Death Dis. 2026 Jun 11;17(1):736. doi: 10.1038/s41419-026-08876-x (PMC13478633; doi:10.1038/s41419-026-08876-x)
Supplement: Supplementary file 6 [file 41419_2026_8876_MOESM2_ESM.pdf]

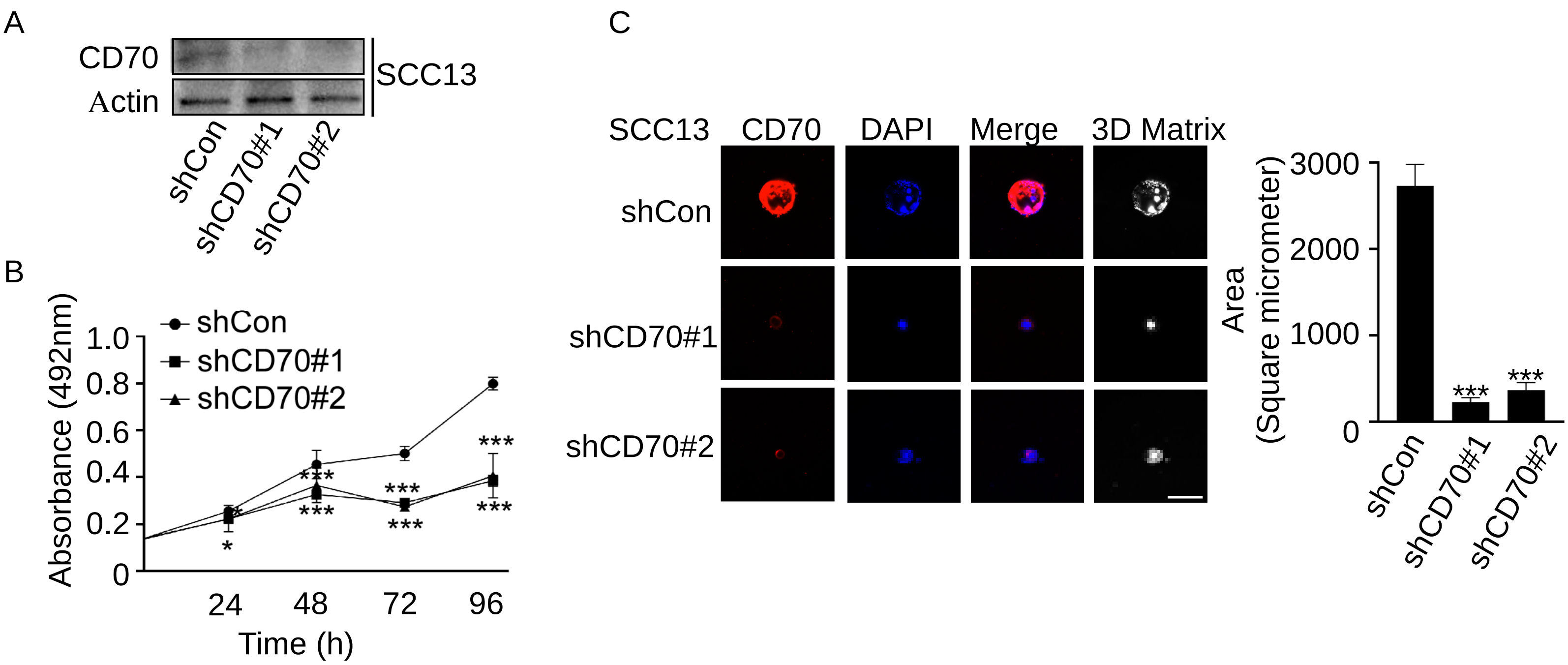

A
CD70
Actin
SCC13
shCon
shCD70#1
shCD70#2
B
Absorbance (492nm)
1.0
0.8
0.6
0.4
0.2
0
shCon
shCD70#1
shCD70#2
24
48
72
96
Time (h)
C
SCC13
CD70
DAPI
Merge
3D Matrix
shCon
shCD70#1
shCD70#2
Area
(Square micrometer)
3000
2000
1000
0
