# Supplementary material for: CD70 drives cSCC growth by linking DNA damage response, inflammation, and tumor–stromal signaling
Source: Cell Death Dis. 2026 Jun 11;17(1):736. doi: 10.1038/s41419-026-08876-x (PMC13478633; doi:10.1038/s41419-026-08876-x)
Supplement: Supplementary file 7 [file 41419_2026_8876_MOESM3_ESM.pdf]

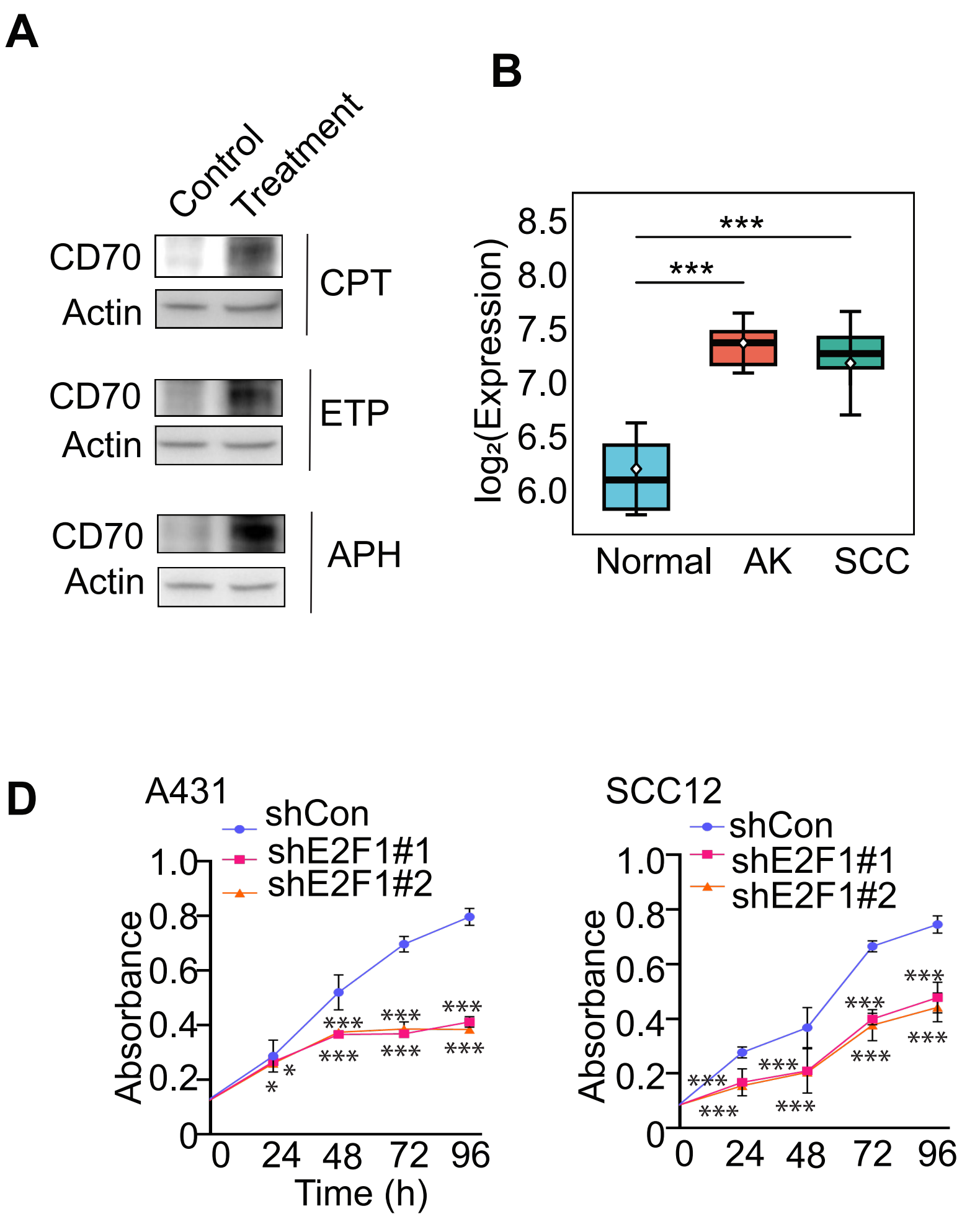
A
Control
Treatment
CD70
Actin
CPT
CD70
Actin
ETP
CD70
Actin
APH
B
log₂(Expression)
8.5
8.0
7.5
7.0
6.5
6.0
***
***
Normal
AK
SCC
C
A431
shCon
shE2F1
#1
#2
E2F1
CD70
Actin
SCC12
shCon
shE2F1
#1
#2
E2F1
CD70
Actin
D
A431
shCon
shE2F1#1
shE2F1#2
Absorbance
1.0
0.8
0.6
0.4
0.2
0
0
24
48
72
96
Time (h)
SCC12
shCon
shE2F1#1
shE2F1#2
Absorbance
1.0
0.8
0.6
0.4
0.2
0
0
24
48
72
96
