# Supplementary material for: CD70 drives cSCC growth by linking DNA damage response, inflammation, and tumor–stromal signaling
Source: Cell Death Dis. 2026 Jun 11;17(1):736. doi: 10.1038/s41419-026-08876-x (PMC13478633; doi:10.1038/s41419-026-08876-x)
Supplement: Supplementary file 8 [file 41419_2026_8876_MOESM4_ESM.pdf]

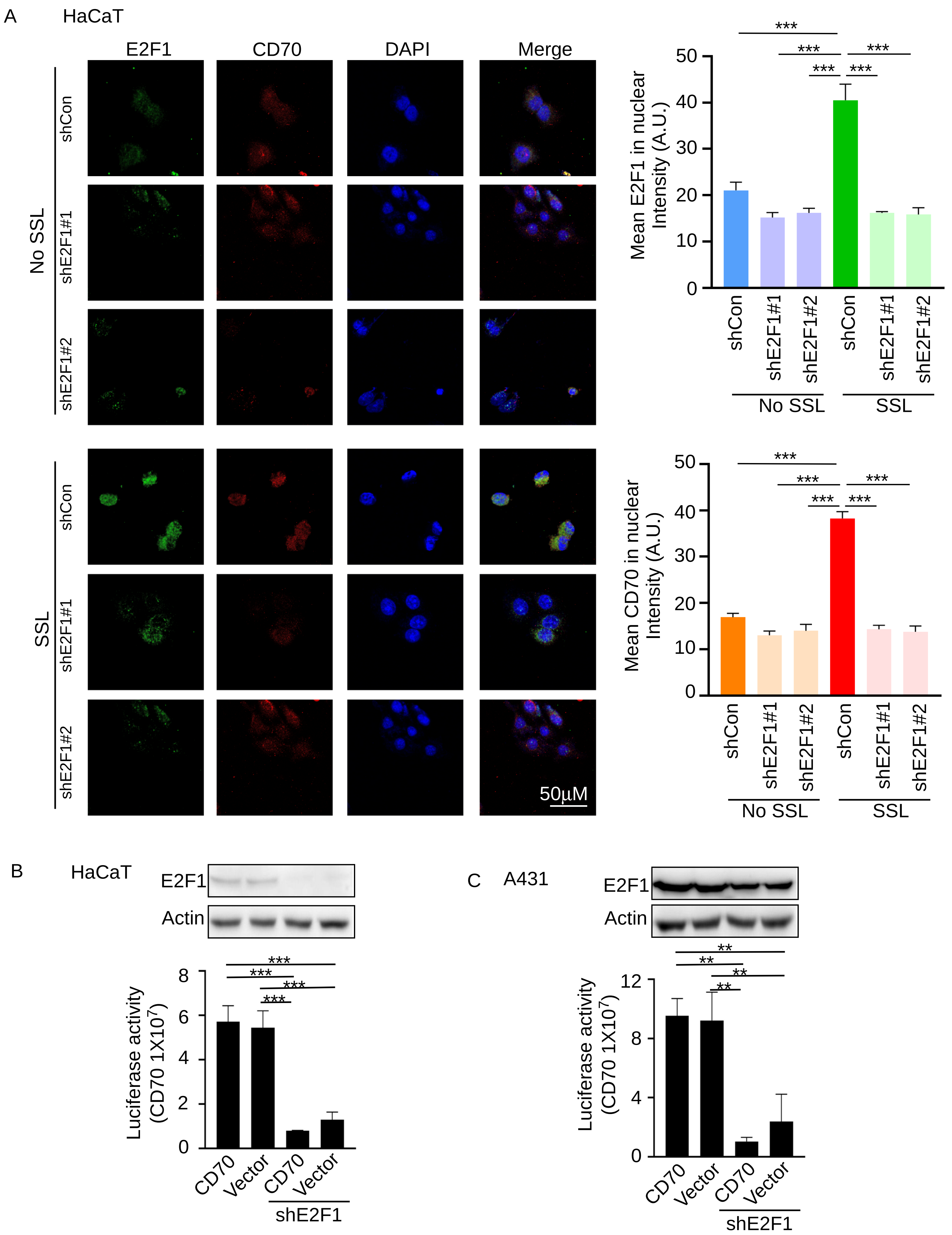

A
HaCaT
E2F1
CD70
DAPI
Merge
No SSL
SSL
shCon
shE2F1#1
shE2F1#2
50μM
Mean E2F1 in nuclear Intensity (A.U.)
Mean CD70 in nuclear Intensity (A.U.)
0
10
20
30
40
50
***
B
HaCaT
E2F1
Actin
Luciferase activity (CD70 1X10^7)
0
2
4
6
8
CD70
Vector
shE2F1
C
A431
12
**
