# Supplementary material for: CD70 drives cSCC growth by linking DNA damage response, inflammation, and tumor–stromal signaling
Source: Cell Death Dis. 2026 Jun 11;17(1):736. doi: 10.1038/s41419-026-08876-x (PMC13478633; doi:10.1038/s41419-026-08876-x)
Supplement: Supplementary file 9 [file 41419_2026_8876_MOESM5_ESM.pdf]

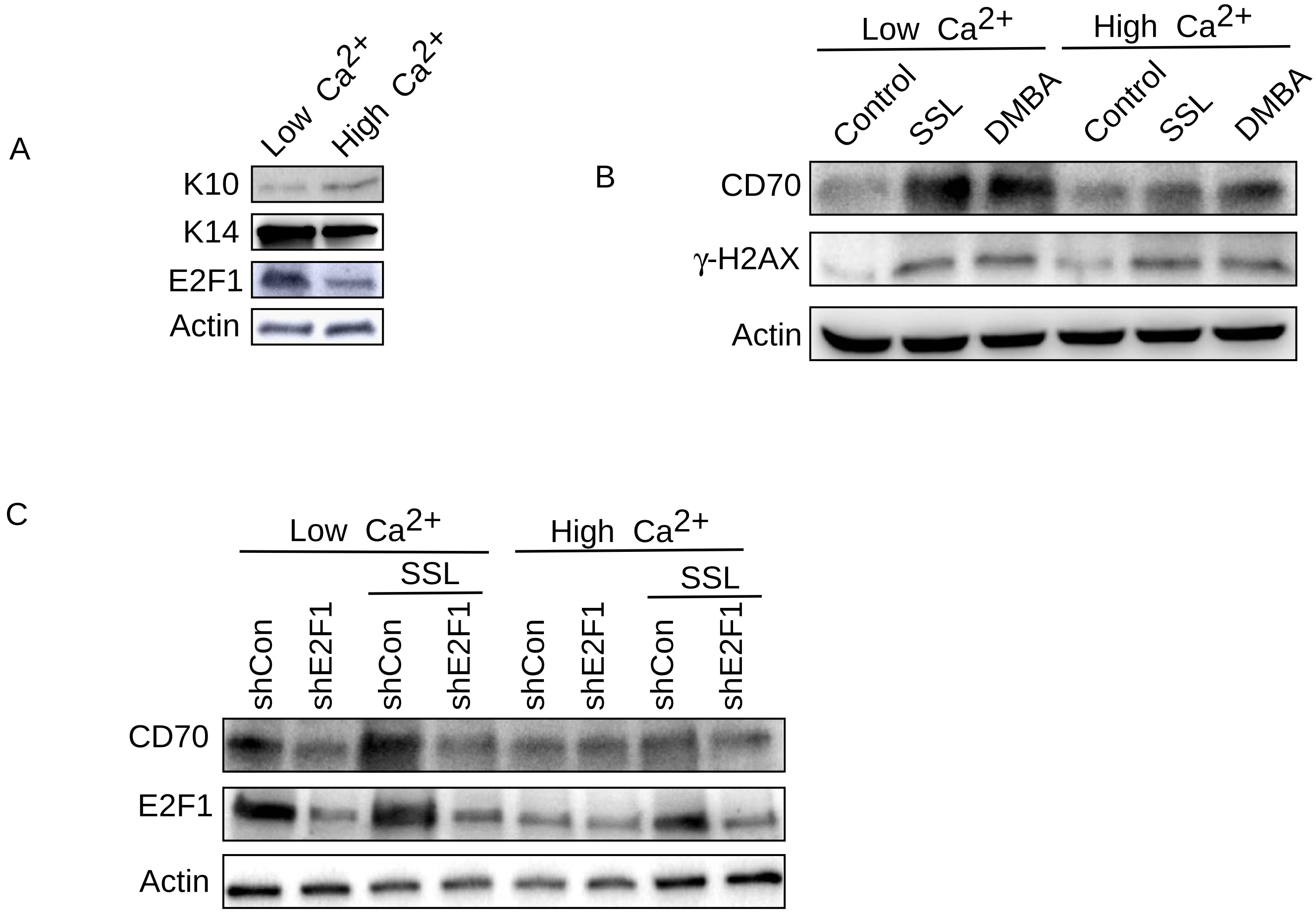
A
Low Ca2+
High Ca2+
K10
K14
E2F1
Actin
B
Low Ca2+
High Ca2+
Control
SSL
DMBA
Control
SSL
DMBA
CD70
γ-H2AX
Actin
C
Low Ca2+
High Ca2+
SSL
SSL
shCon
shE2F1
shCon
shE2F1
shCon
shE2F1
shCon
shE2F1
CD70
E2F1
Actin
