# Supplementary material for: CD70 drives cSCC growth by linking DNA damage response, inflammation, and tumor–stromal signaling
Source: Cell Death Dis. 2026 Jun 11;17(1):736. doi: 10.1038/s41419-026-08876-x (PMC13478633; doi:10.1038/s41419-026-08876-x)
Supplement: Supplementary file 10 [file 41419_2026_8876_MOESM6_ESM.pdf]

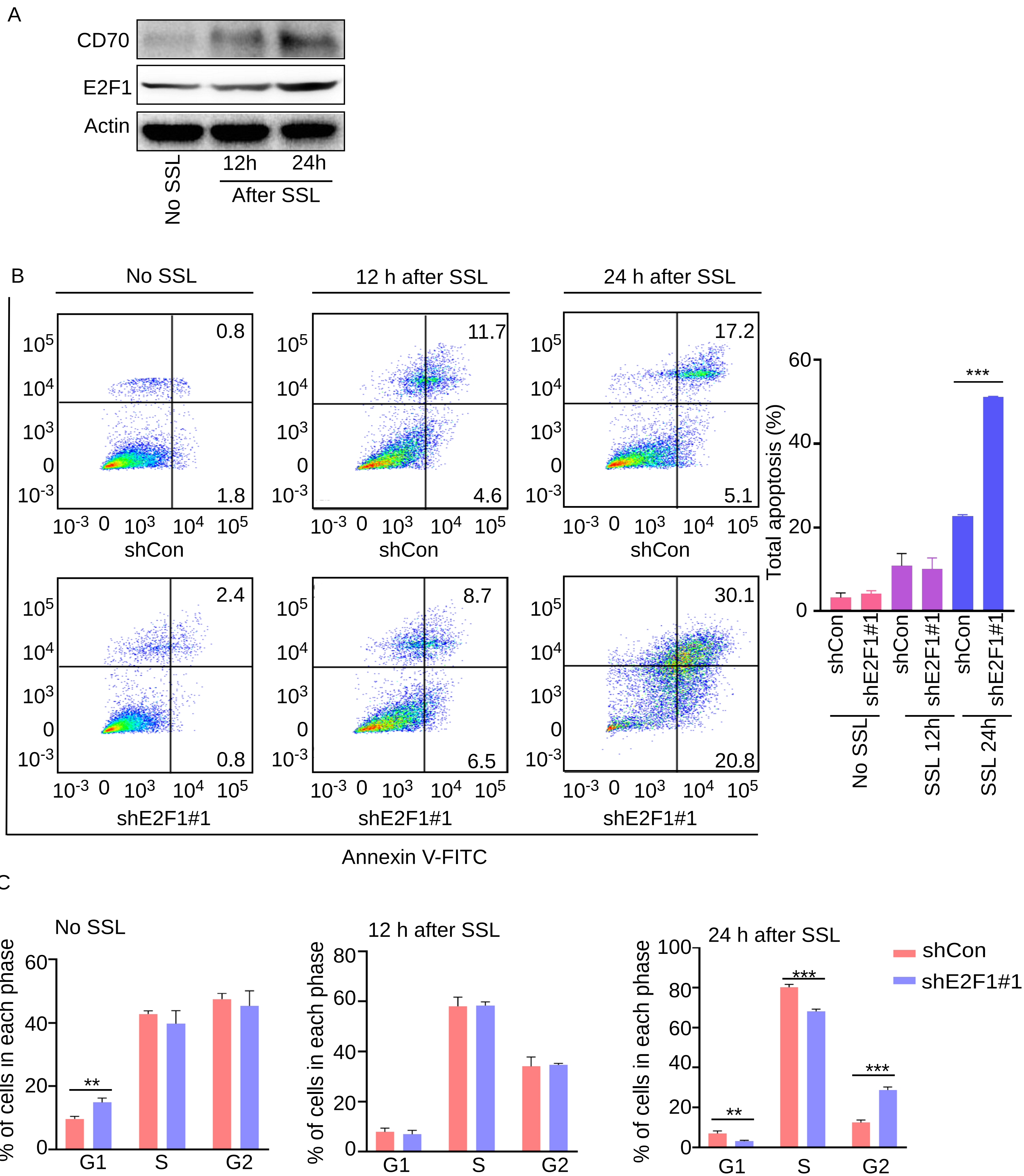

A
CD70
E2F1
Actin
No SSL
12h
24h
After SSL
B
No SSL
12 h after SSL
24 h after SSL
Propidium iodide
0.8
1.8
11.7
4.6
17.2
5.1
2.4
0.8
8.7
6.5
30.1
20.8
shCon
shE2F1#1
Annexin V-FITC
Total apoptosis (%)
***
No SSL
SSL 12h
SSL 24h
C
No SSL
12 h after SSL
24 h after SSL
% of cells in each phase
G1
S
G2
**
***
shCon
shE2F1#1
