# Supplementary material for: CD70 drives cSCC growth by linking DNA damage response, inflammation, and tumor–stromal signaling
Source: Cell Death Dis. 2026 Jun 11;17(1):736. doi: 10.1038/s41419-026-08876-x (PMC13478633; doi:10.1038/s41419-026-08876-x)
Supplement: Supplementary file 11 [file 41419_2026_8876_MOESM7_ESM.pdf]

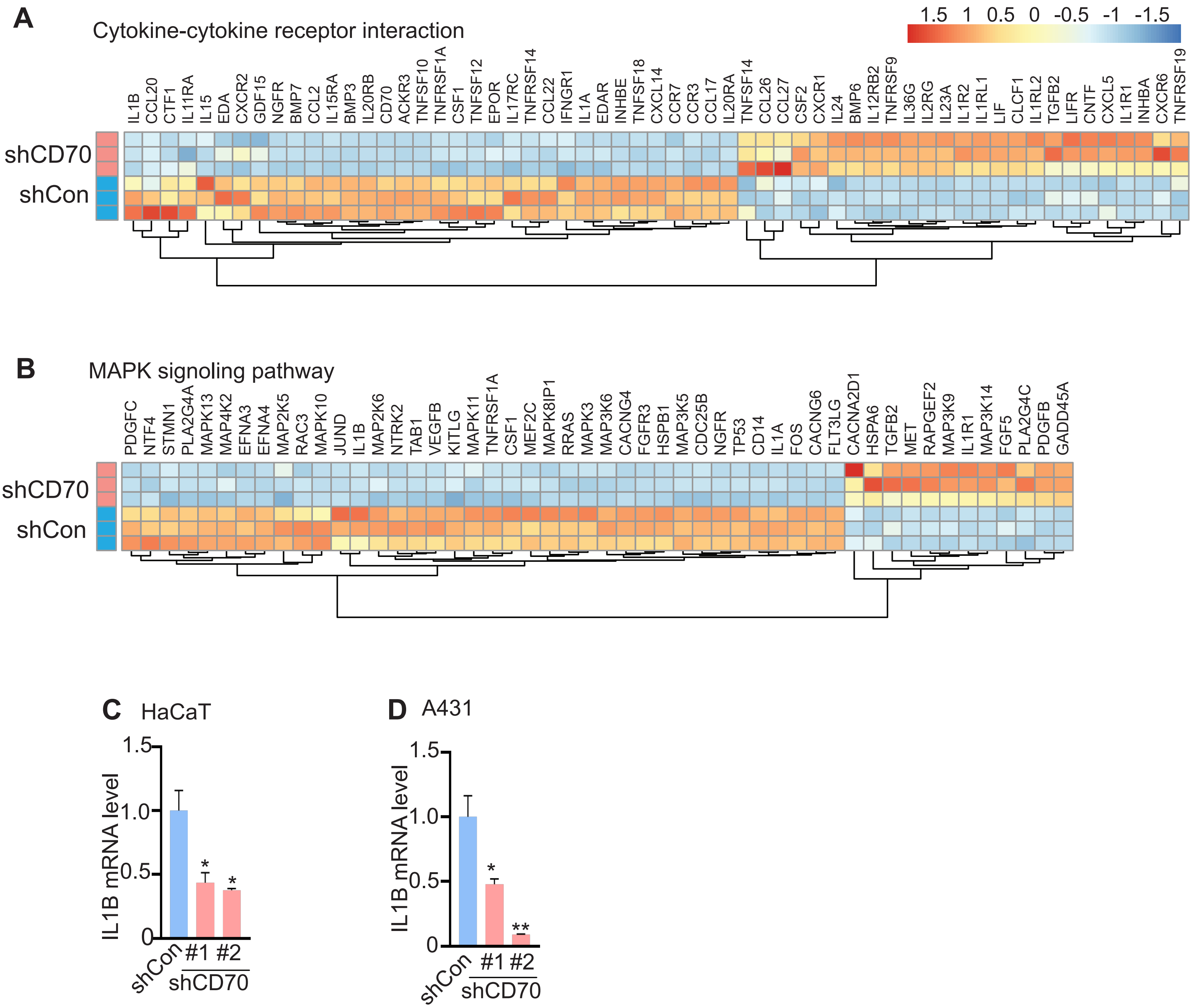
A
Cytokine-cytokine receptor interaction
1.5 1 0.5 0 -0.5 -1 -1.5
IL1B CCL20 CTF1 IL11RA IL15 EDA CXCR2 GDF15 NGFR BMP7 CCL2 IL15RA BMP3 IL20RB CD70 ACKR3 TNFSF10 TNFRSF1A CSF1 TNFSF12 EPOR IL17RC TNFRSF14 CCL22 IFNGR1 IL1A EDAR INHBE TNFSF18 CXCL14 CCR7 CCR3 CCL17 IL20RA TNFSF14 CCL26 CCL27 CSF2 CXCR1 IL24 BMP6 IL12RB2 TNFRSF9 IL36G IL2RG IL23A IL1R2 IL1RL1 LIF CLCF1 IL1RL2 TGFB2 LIFR CNTF CXCL5 IL1R1 INHBA CXCR6 TNFRSF19
shCD70
shCon
B
MAPK signoling pathway
PDGFC NTF4 STMN1 PLA2G4A MAPK13 MAP4K2 EFNA3 EFNA4 MAP2K5 RAC3 MAPK10 JUND IL1B MAP2K6 NTRK2 TAB1 VEGFB KITLG MAPK11 TNFRSF1A CSF1 MEF2C MAPK8IP1 RRAS MAPK3 MAP3K6 CACNG4 FGFR3 HSPB1 MAP3K5 CDC25B NGFR TP53 CD14 IL1A FOS CACNG6 FLT3LG CACNA2D1 HSPA6 TGFB2 MET RAPGEF2 MAP3K9 IL1R1 MAP3K14 FGF5 PLA2G4C PDGFB GADD45A
shCD70
shCon
C HaCaT
IL1B mRNA level
1.5
1.0
0.5
0
*
*
shCon
#1 #2
shCD70
D A431
IL1B mRNA level
1.5
1.0
0.5
0
*
**
shCon
#1 #2
shCD70
