# Supplementary material for: CD70 drives cSCC growth by linking DNA damage response, inflammation, and tumor–stromal signaling
Source: Cell Death Dis. 2026 Jun 11;17(1):736. doi: 10.1038/s41419-026-08876-x (PMC13478633; doi:10.1038/s41419-026-08876-x)
Supplement: Supplementary file 12 [file 41419_2026_8876_MOESM8_ESM.pdf]

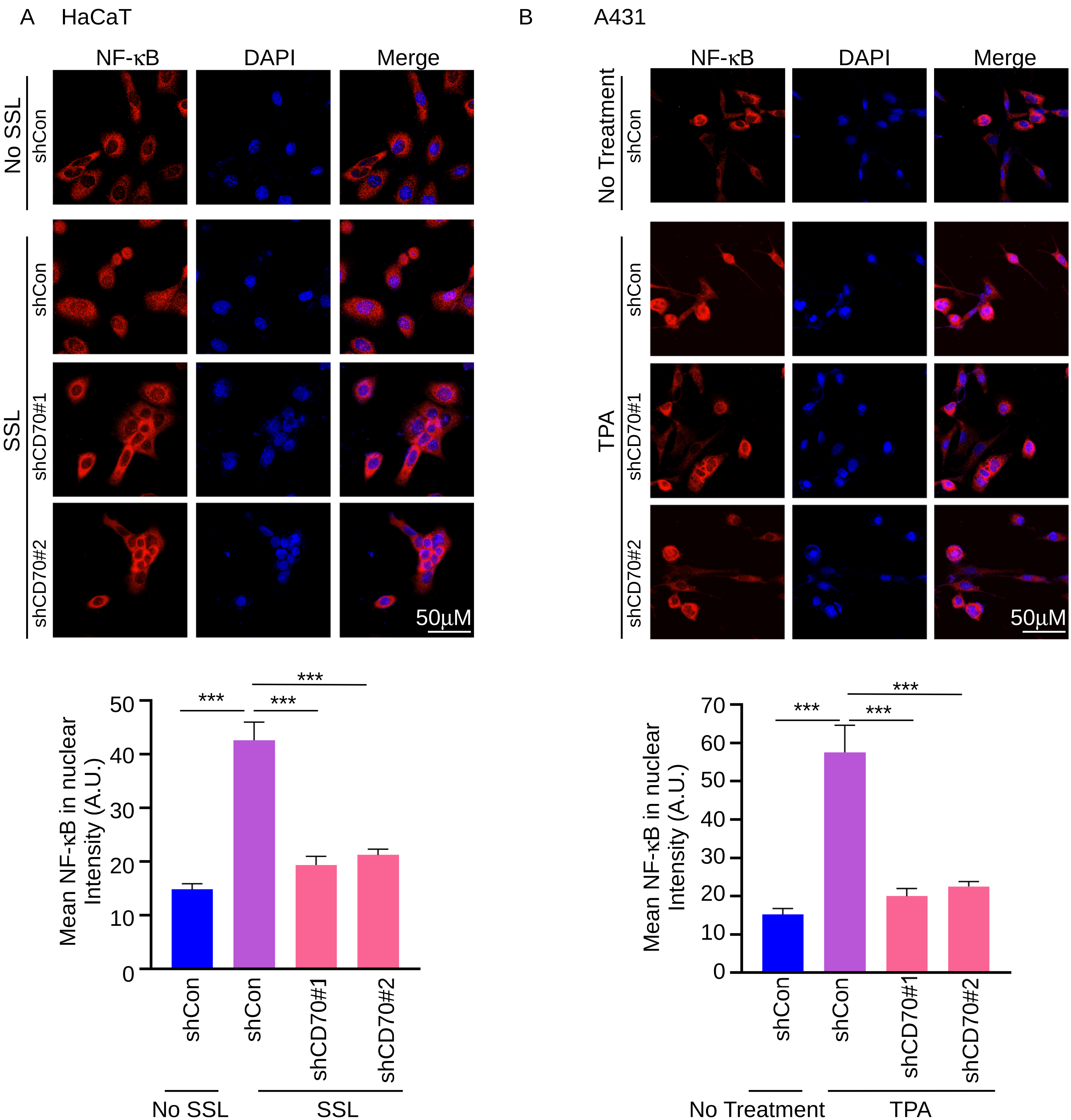

A
HaCaT
NF-κB
DAPI
Merge
No SSL
shCon
SSL
shCon
shCD70#1
shCD70#2
50μM
Mean NF-κB in nuclear Intensity (A.U.)
0
10
20
30
40
50
***
***
***
shCon
shCon
shCD70#1
shCD70#2
No SSL
SSL
B
A431
NF-κB
DAPI
Merge
No Treatment
shCon
TPA
shCon
shCD70#1
shCD70#2
50μM
Mean NF-κB in nuclear Intensity (A.U.)
0
10
20
30
40
50
60
70
***
***
***
shCon
shCon
shCD70#1
shCD70#2
No Treatment
TPA
