# Supplementary material for: CD70 drives cSCC growth by linking DNA damage response, inflammation, and tumor–stromal signaling
Source: Cell Death Dis. 2026 Jun 11;17(1):736. doi: 10.1038/s41419-026-08876-x (PMC13478633; doi:10.1038/s41419-026-08876-x)
Supplement: Supplementary file 13 [file 41419_2026_8876_MOESM9_ESM.pdf]

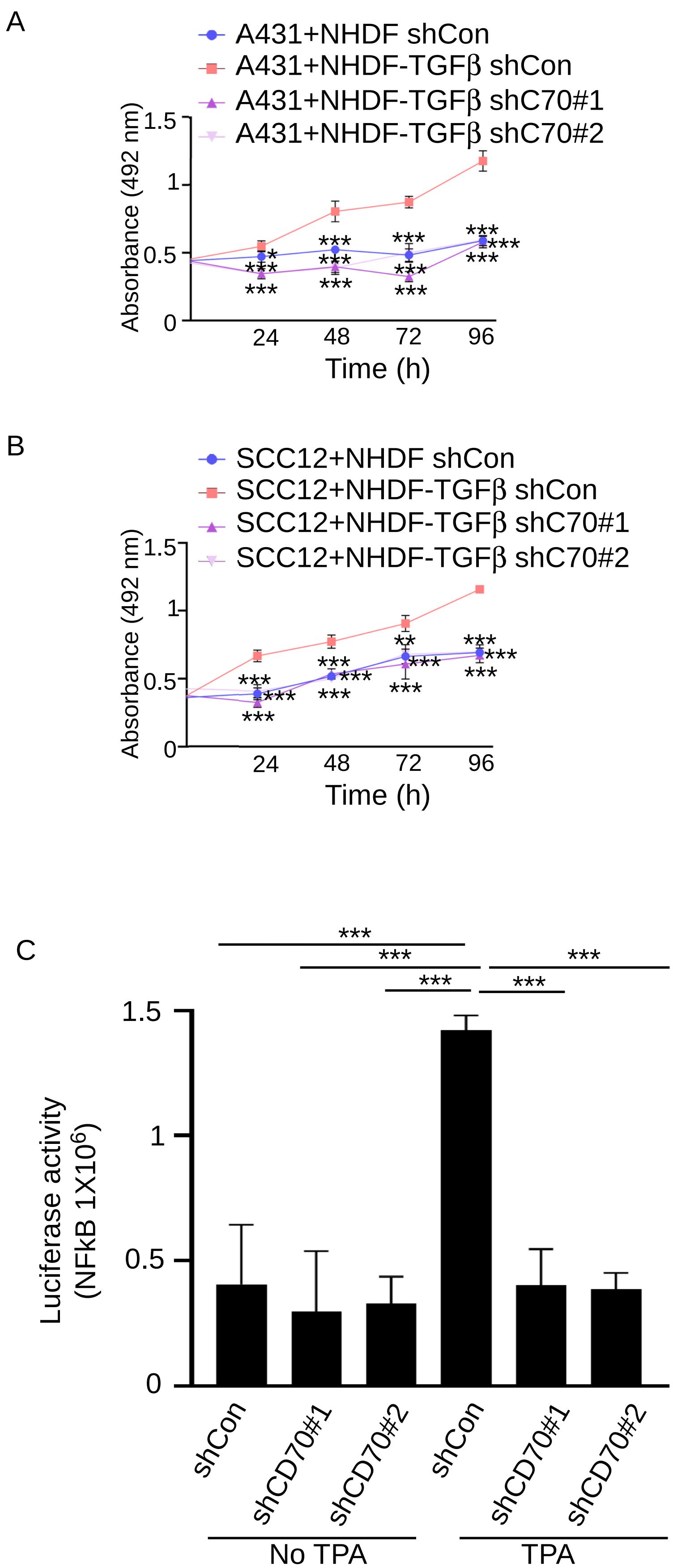

A
A431+NHDF shCon
A431+NHDF-TGFβ shCon
A431+NHDF-TGFβ shC70#1
A431+NHDF-TGFβ shC70#2
Absorbance (492 nm)
0
0.5
1
1.5
24
48
72
96
Time (h)
B
SCC12+NHDF shCon
SCC12+NHDF-TGFβ shCon
SCC12+NHDF-TGFβ shC70#1
SCC12+NHDF-TGFβ shC70#2
Absorbance (492 nm)
0
0.5
1
1.5
24
48
72
96
Time (h)
C
Luciferase activity
(NFkB 1X10^6)
0
0.5
1
1.5
shCon
shCD70#1
shCD70#2
shCon
shCD70#1
shCD70#2
No TPA
TPA
