# Supplementary material for: CD70 drives cSCC growth by linking DNA damage response, inflammation, and tumor–stromal signaling
Source: Cell Death Dis. 2026 Jun 11;17(1):736. doi: 10.1038/s41419-026-08876-x (PMC13478633; doi:10.1038/s41419-026-08876-x)
Supplement: Supplementary file 14 [file 41419_2026_8876_MOESM12_ESM.docx]

**Supplementary Figure Legends**

**Figure S1. The expression of CXCL13 and CD70 in non-melanoma skin cancers. (A)** CXCL13 expression is significantly lower in sun-exposed skin compared with sun-protected skin (left panel) but is markedly elevated in AK and cSCC compared with normal controls (right panel). **(B, C)** CD70 expression in SCC stratified by tumor stage and grade (SK801c, US Biomax). Statistical analysis was performed using the Wilcoxon rank-sum test (****p* < 0.001).

**Figure S2. Knockdown of CD70 suppresses cSCC cell growth in SCC13 cells. (A)** SCC13 cells with stable shCon or shCD70 knockdown were established, and CD70 expression was confirmed by Western blotting. **(B)** CD70 knockdown reduces the proliferation of SCC13 cells as measured by MTS assays. **(C)** CD70 knockdown decreases spheroid growth of SCC13 cells in 3D spheroid assays. CD70 is shown in red, and nuclei are counterstained with DAPI (blue). Scale bar: 50 µm. Asterisks indicate statistical significance (**p* < 0.05; ****p* < 0.001 vs. shCon in each time point; Student’s *t*-test).

**Figure S3. DNA damage response mediates CD70 expression *via* E2F1. (A)** Treatment of HaCaT cells with DNA-damaging agents (CPT, ETP, or APH) for 24 h enhances CD70 expression. **(B)** E2F1 expression is markedly elevated in AK and cSCC compared with normal controls. Statistical analysis was performed using Wilcoxon rank-sum test (****p* < 0.001). Knockdown of E2F1 significantly decreases CD70 expression **(C)** and suppresses growth **(D)** in A431 and SCC12 cells. Asterisks indicate statistical significance (**p* < 0.05; ****p* < 0.001 vs. shCon; Student’s *t*-test).

**Figure S4. Knockdown of E2F1 suppresses SSL-induced CD70 expression in HaCaT cells. (A)** HaCaT cells expressing shCon or shE2F1 were generated, and E2F1 and CD70 expression was assessed by immunofluorescence (upper panels). Scale bar: 50 μm. **(B, C)** Luciferase assays show that E2F1 overexpression enhances CD70 promoter activity in **(B)** HaCaT and **(C)** A431 cells. Fluorescence intensity was quantified using ZEISS ZEN 3.7 (lower panels). Statistical significance was determined by one-way ANOVA with Tukey’s HSD (***p* < 0.01; ****p* < 0.001).

**Figure S5. E2F1 regulates CD70 expression in primary human keratinocytes under differentiation conditions.** **(A)** Expression of K10, K14, and E2F1 in primary human keratinocytes cultured under low- and high-calcium conditions. Differentiation was induced by treatment with 1.5 mM CaCl₂ for 48 h. **(B)** Induction of CD70 and γH2AX by SSL or DMBA in primary human keratinocytes under low- and high-calcium conditions. **(C)** E2F1 knockdown reduces CD70 expression under both low- and high-calcium conditions.

**Figure S6. E2F1 regulates CD70 expression independently of apoptosis and cell cycle changes.** **(A)** Western blot analysis of E2F1 and CD70 expression in HaCaT cells at 12 h and 24 h following SSL exposure. **(B)** Apoptosis analysis of control and E2F1-knockdown HaCaT cells under basal conditions and after SSL exposure, assessed by Annexin V/PI staining. **(C)** Cell cycle analysis of control and E2F1-knockdown HaCaT cells under basal conditions and after SSL exposure, determined by PI staining. Asterisks indicate statistical significance (***p* < 0.01; ****p* < 0.001 vs. shCon in each time point; Student’s *t*-test).

**Figure S7. Knockdown of CD70 mediates cytokine-cytokine receptor interaction and MAPK signaling pathways.** KEGG analysis highlights cytokine–cytokine receptor interaction and MAPK signaling as the most altered pathways (see Figure 4C). Heatmap analysis shows the detailed gene expression changes in the cytokine–cytokine receptor interaction **(A)** and MAPK signaling pathways **(B)**. To validate these results, CD70 knockdown results in decreased *IL1B* expression, one of the common genes shared by both pathways, as determined by RT-qPCR in SSL-exposed HaCaT cells **(C)** and A431 cells **(D)**. Asterisks indicate statistical significance (**p* < 0.05; ***p* < 0.01 vs. shCon; Student’s *t*-test).

**Figure S8. Knockdown of CD70 suppresses NF-κB nuclear translocation in keratinocytes and cSCC cells.** HaCaT **(A)** and A431 **(B)** cells expressing shCon or shCD70 were established. IF staining shows that CD70 knockdown reduces SSL-induced NF-κB nuclear translocation in HaCaT cells and TPA-induced NF-κB nuclear translocation in A431 cells (upper panels). Scale bar: 50 μm. Nuclear fluorescence intensity was quantified using ZEISS ZEN 3.7 (lower panels). Data are presented as mean values ± SE. Statistical significance was determined by one-way ANOVA with Tukey’s HSD (****p* < 0.001).

**Figure S9. Conditioned medium (CM) from CD70-silenced fibroblasts suppresses cSCC cell growth and CD70-silenced suppresses TPA-induced NK-κB activation in** NHDF1-TGFβ cells**. (A)** MTT assay showing that CM from NHDF1-TGFβ shCon fibroblasts enhance A431 cell growth, whereas CM from NHDF1-TGFβ shCD70 fibroblasts suppresses A431 growth compared with shCon CM. **(B)** Similar results were observed in SCC12 cells. Here CM from NHDF1-TGFβ shCon fibroblasts promotes growth while CM from NHDF1-TGFβ shCD70 fibroblasts suppresses growth. Data are presented as means ± SE, with statistical significance determined by Student’s *t*-test (****p* < 0.001 vs. NHDF1-TGFβ shCon CM). **(C)** Knockdown of CD70 suppresses NF-κB activation with TPA treatment in NHDF1-TGFβ cells. Statistical significance was determined by one-way ANOVA with Tukey’s HSD (****p* < 0.001).

**Figure S10. CD70 mediates cytokine secretion from NHDF1-TGFβ fibroblasts.** Cytokine levels in conditioned medium (CM) from NHDF1-TGFβ fibroblasts expressing shCD70 or shCon were assessed using a human cytokine array. Spot intensities were quantified using ImageJ, and cytokine expression levels are presented in graphical form. IL-6 and MCP-3 are two examples of cytokines that are markedly altered between shCD70 and shCon fibroblasts, highlighting CD70’s role in regulating pro-inflammatory cytokine secretion.

**Figure S11. CD70 mediates NF-κB activation and tumor-promoting function in NHDF-TGFβ fibroblasts. (A)** NF-κB activation in control, CD70-knockdown, and CD70 re-expressing NHDF-TGFβ cells. **(B)** NF-κB nuclear localization in NHDF-TGFβ cells following CD70 knockdown or re-expression. **(C)** A431 spheroid growth co-cultured with NHDF-TGFβ cells with CD70 knockdown or re-expression. Statistical significance was determined by one-way ANOVA with Tukey’s HSD (***p* < 0.01; ****p* < 0.001).
