# Supplementary material for: CD70 drives cSCC growth by linking DNA damage response, inflammation, and tumor–stromal signaling
Source: Cell Death Dis. 2026 Jun 11;17(1):736. doi: 10.1038/s41419-026-08876-x (PMC13478633; doi:10.1038/s41419-026-08876-x)
Supplement: Supplementary file 15 [file 41419_2026_8876_MOESM13_ESM.docx]

**Supplementary Materials and Methods**

**Reagents and antibodies**

For cell culture, Dulbecco’s Modified Eagle Medium (DMEM), gentamicin, and penicillin were obtained from Invitrogen (Carlsbad, CA), while MEM non-essential amino acids were purchased from Corning (Corning, NY). Fetal bovine serum (FBS) was obtained from Gemini Bio-Products (West Sacramento, CA). Additional laboratory reagents, including Tris, NaCl, sodium bicarbonate, hydrocortisone, glucose, transferrin, deoxyribonuclease I, trypsin-EDTA solution (T4049), SDS, transforming growth factor-β (TGF-β; #616451), trichloroacetic acid (TCA; T9159-250G), and acetone (904082-2.5L) were purchased from Sigma-Aldrich (St. Louis, MO). Aphidicolin (APH, 14007), camptothecin (CPT, 11694), and etoposide (ETP, 12092) were purchased from Cayman Chemical (Ann Arbor, MI); and 7,12-dimethylbenz[a]anthracene (DMBA, 57-97-6) and calcium chloride dihydrate (C7902-500G) were purchased from Millipore-Sigma (Burlington, MA). Caffeine (58-08-2, Cat#33898) was purchased from Chem-Impex International (Wood Dale, IL). Primary antibodies used in this study included β-actin (sc-47778), CD70 (sc-365539), Cylin A (sc-596), Cyclin E (sc-248) MCP-3 (sc-515890), and IL-6 (sc-130326) (all from Santa Cruz Biotechnology, Dallas, TX); phospho-PI3K (#4228), PI3K (#5405), phospho-AKT (#9271), AKT (#9272), phospho-ERK (#9101), ERK (#9102), phospho-JNK (#9251), JNK (#9252), phospho-p38 (#4511), p38 (#8690), E2F1 (#3742), NF-κB p65 (#8242), PCNA (#13110), and vimentin (#5741) (all from Cell Signaling Technology, Danvers, MA); γ-H2AX (613402), pan-cytokeratin (628606), keratin 10 (Cat#905404), and keratin 14 (Cat#905303) were purchased from BioLegend (San Diego, CA). For Western blotting, TrueBlot Ultra secondary antibodies were used and included anti-rabbit IgG HRP (18-8816-31) and anti-mouse IgG HRP (18-8817-31) (Rockland Immunochemicals, Limerick, PA). For immunofluorescence (IF), Alexa Fluor–conjugated secondary antibodies anti-rabbit (A32740, A27034) and anti-mouse (A11005) were purchased from Invitrogen. Human Interleukin-6 ELISA Kit (Item No. 501030) was purchased from Cayman Chemical (Ann Arbor, MI).

**Transcriptomic data collection and analysis**

The RNA expression data from sun exposed and sun protected human skin were obtained from the Genotype-Tissue Expression (GTEx) database. Microarray datasets (GSE2503, GSE42677) were retrieved from the Gene Expression Omnibus (GEO) and merged using the R package inSilicoMerging, with batch effects corrected using the Empirical Bayes method. Differentially expressed genes (DEGs) between sun protected and sun exposed skin, and between actinic keratosis (AK) or squamous cell carcinoma (SCC) and normal skin, were identified using the limma package (v3.58.1) in R. Expression data were log2-transformed, and a design matrix was constructed according to sample group assignments. Linear modeling was performed using the lmFit function, followed by empirical Bayes moderation (eBayes). Genes with an FDR-adjusted *p*-value < 0.05 and |log2 fold change| ≥ 1 were considered significantly differentially expressed. Gene expression profiles of CD70, CXCL13, and E2F1 were extracted from GTEx and GEO datasets. Box plots were generated to display expression distributions across groups, with mean values indicated as points and error bars representing means ± standard deviation.

Total RNA from HaCaT cells 24 h post solar-simulated light (SSL; 60 kJ/m² UVA, 2.9 kJ/m² UVB) exposure was extracted using the RNeasy Mini QIAcube Kit (QIAGEN, Germantown, MD). Library preparation and sequencing were performed by LC Sciences (Houston, TX); Differentially Expressed Genes (DEGs) between shCon and shCD70 groups were identified following the same procedure as above, with linear modeling, contrast fitting, and empirical Bayes moderation. Volcano plots were generated with ggplot2 (v3.5.1), highlighting upregulated DEGs in red, downregulated DEGs in blue, and non-significant genes in gray.

KEGG pathway enrichment analysis was conducted to identify biological pathways significantly associated with DEGs. Enrichment results were visualized using bubble plots, with gene ratio on the x-axis, pathways on the y-axis, bubble size representing gene count, and color representing -log10 (FDR).

Heatmaps were generated from normalized RNA-seq expression data (FPKM values) by using pheatmap and tidyverse in R. Samples were annotated by experimental group (“shCon” and “shCD70”), and row-wise Z-score normalization was applied to standardize gene expression levels. Hierarchical clustering was performed for both genes and samples to reveal co-expression patterns. This approach was applied to visualize genes in the cytokine-cytokine receptor interaction and MAPK signaling pathways, providing a comparative view of expression changes between control and CD70-knockdown conditions.

**RNA extraction library construction and sequencing**

Total RNA was extracted using the RNeasy Mini QIAcube Kit (QIAGEN, 19300 Germantown, MD) following the manufacturer's instructions and purity was checked by using the Bioanalyzer 2100 and RNA 6000 Nano LabChip Kit (Agilent, Santa Clara, CA). After total RNA extraction and purification, mRNA was extracted using Dynabeads Oligo (dT) (Thermo Fisher, Waltham, CA). Following purification, the mRNA was fragmented using the Magnesium RNA Fragmentation Module (Cat. e6150, NEB, Ipswich, MA). From the RNA fragments, cDNA by SuperScript™ II Reverse Transcriptase (Cat. 1896649, Invitrogen, Carlsbad, CA) was synthesized. Dual-index adapters were ligated to the fragments, and size selection was performed with Amperex beads. After the heat-labile UDG enzyme (Cat. m0280, NEB) treatment of the U-labeled second-stranded DNAs, the ligated products were amplified with PCR. We then performed the 2×150 bp paired-end sequencing (PE150) on an Illumina Novaseq™ 6000 following the vendor's recommended protocol. The RNA sequencing was performed by LC Science (Houston, TX).

**Bioinformatics analysis for RNAseq**

*Sequence and filtering of clean reads*

A cDNA library was constructed using pooled RNA from mouse skin samples and sequencing was performed using the Illumina NovaseqTM 6000 sequence platform. Using the Illumina paired-end RNA-seq approach, we sequenced the transcriptome, generating a total of 2 million x 150 bp paired-end reads. Reads obtained from the sequencing analysis included adapters or low-quality bases that may have the potential to affect the assembly and analysis. Thus, to obtain high quality clean reads, reads were further filtered by Cutadapt (1). The parameters were as follows: 1) removing reads containing adapters; 2) removing reads containing polyA and polyG; 3) removing reads containing more than 5% of unknown nucleotides (N); and 4) removing low quality reads containing more than 20% of low quality (Q-value ≤20) bases including the Q20, Q30 and GC-content of the clean data. Following that procedure, a total of gigabase pairs (G bp) of cleaned, paired-end reads was produced.

*Alignment with reference genome*

We aligned reads of all samples to the < research species > reference genome by using the HISAT2 (version: hisat2-2.0.4) package, which initially removes a portion of the reads based on quality information accompanying each read and then maps the reads to the reference genome. HISAT2 allows multiple alignments per read (up to 20 by default) and a maximum of two mismatches when mapping the reads to the reference. HISAT2 builds a database of potential splice junctions and confirms these by comparing the previously unmapped reads against the database of putative junctions (2-4).

*Quantification of gene abundance*

The mapped reads of each sample were assembled using StringTie (version: stringtie-1.3.4d) with default parameters. Then, all transcriptomes from all samples were merged to reconstruct a comprehensive transcriptome by using gffcompare software ([version:gffcompare-0.9.8](http://ccb.jhu.edu/software/stringtie/gffcompare.shtml,version:gffcompare-0.9.8)) After the final transcriptome was generated, StringTie and ballgown were used to estimate the expression levels of all transcripts and perform expression abundance for mRNAs by calculating FPKM (fragment per kilobase of transcript per million mapped reads) value (4-6).

*Differentially expressed gene analysis*

We utilized the R package Limma (version 3.40.6) for differential analysis, aiming to identify differences in gene expression between the treatment and control groups. The genes with a parameter of *p* value below 0.05 and absolute fold change ≥ 2 were considered differentially expressed genes. Then we performed the enrichment analysis (KEGG) by using the National Institutes of Health DAVID Bioinformatics software.

**Reverse phase microarray analysis (RPPA)**

Lysates were prepared and printed in triplicate spots (approx. 10nL per spot) onto nitrocellulose coated slides (Grace Biolabs, Bend, OR) using a Quanterix 2470 Arrayer (Quanterix, Billerica, MA). Standard curves of control cell lysates were included for quality assurance purposes. CD70 Antibody used on the RPPA were validated before use by confirming the presence of a single band at the appropriate molecular weight with a panel of control cell lysates using conventional western blotting. Immunostaining was performed by probing each slide with one primary antibody targeting the protein of interest. Biotinylated goat anti-rabbit IgG (H+L) (1:7,500, Vector Laboratories Inc, Burlingame, CA) or rabbit anti-mouse IgG (1:10, DakoCytomation, Carpinteria, CA) were used as secondary antibodies. Signal amplification was performed using a tyramide-based avidin/biotin amplification system (DakoCytomation, Carpinteria, CA) followed by streptavidin-conjugated IRDye 680 (LI-COR, Lincoln, NE) for visualization. Negative controls were stained with secondary antibody alone. Total protein was measured using Sypro Ruby protein blot staining per manufacturer’s instructions (Molecular Probes, Eugene, OR). RPPA data were generated directly from images acquired using a Tecan PowerScanner (Tecan, Mannedorf, Switzerland) and analyzed with MicroVigene software Version 5.1.0.0 (Vigenetech, Carlisle, MA). Total protein intensities for each sample were calculated by averaging the Sypro staining intensity of the three replicate spots. For each sample/endpoint the final signal intensity was calculated by: 1) subtraction of negative control spot intensity from primary antibody spot intensity, 2) averaging the resulting net intensities for the three replicate spots, and 3) dividing by the total protein intensity value for each sample.

**Western blot analysis**

Western blotting was performed following previously established protocols (7). Protein lysates from cells and conditioned medium were prepared using 1× RIPA lysis buffer supplemented with a protease and phosphatase inhibitor cocktail (R4100-010, Gene Depot, Katy, TX). Protein concentration was determined using a standard curve, and absorbance was measured with the Multiscan MCC Microplate Reader 355 (Thermo Fisher Scientific, Alexandria, VA).

For protein extraction from conditioned medium, 50 g of trichloroacetic acid (TCA) were dissolved in 100 mL of distilled water. A 5-mL sample of conditioned medium was collected, mixed with an equal volume of TCA, vortexed, and incubated on ice for 30 min. The mixture was then centrifuged at 13,000 rpm for 30 min at 4°C. The supernatant fraction was discarded, and the pellet was washed twice with 1 mL of ice-cold acetone. After centrifugation at 13,000 rpm for 10 min at 4°C, the supernatant fraction was removed, and the pellet air-dried. The dried pellet was resuspended in 250 µL of RIPA lysis buffer and subjected to sonication. Protein concentration was again determined using a standard curve with absorbance measured on the Multiscan MCC Microplate Reader 355 (Thermo Fisher Scientific, Alexandria, VA).

Primary antibodies were diluted 1:1000 and incubated overnight at 4°C, followed by incubation with HRP-conjugated secondary antibodies at a 1:5000 dilution. Protein bands were visualized using a chemiluminescent substrate (GE Healthcare Biosciences, Piscataway, NJ).

**Cell viability (MTT) assay**

Cells (A431 and SCC12) expressing shCon or shCD70/shE2F1 were seeded in 96-well plates at a density of 1 × 10⁴ cells per well and allowed to attach overnight. Cell viability was assessed at 24, 48, 72, and 96 h using the MTT assay. Briefly, 20 μL of 5 mg/mL MTT reagent (Sigma-Aldrich) was added to each well and incubated at 37°C for 1 h. Absorbance was measured at 492 nm using a microplate reader (Thermo Fisher Scientific). Data were normalized to the 24-h time point and presented as means ± SE from 3 independent experiments.

To examine the effect of conditioned medium from normal human dermal fibroblasts (NHDF) (shCon or shCD70) on cSCC cell growth, A431 or SCC12 cells were seeded in 96-well plates at 1 × 10⁴ cells per well and allowed to attach overnight. The medium was then replaced with 100 μL of conditioned medium per well, which was refreshed daily. Cell viability was assessed at 24, 48, 72, and 96 h using the MTT assay as described above. Data were normalized to the 24-h time point and presented as means ± SE from 3 independent experiments.

**Immunofluorescence analysis**

For 3D spheroid cultures, the medium was removed from the hydrogel surface, followed by PBS washes. Spheroids were fixed in 4% formaldehyde for 30 min, permeabilized with 0.1% Triton X-100 for 5 min and blocked with 3% BSA for 1 h. Primary antibodies were applied overnight at 4°C, followed by secondary antibodies for 5 h and DAPI counterstaining. Images were acquired with a fluorescence microscope.

For 2D cultures, A431 cells (4 × 10⁴/well) expressing shCon or shCD70, or overexpressing E2F1 were seeded on chamber slides. HaCaT cells (shCon or shE2F1) were seeded separately and exposed to solar-simulated light (60 kJ/m² UVA and 2.9 kJ/m² UVB). After 24 h, cells were fixed in methanol, blocked with BSA, and incubated overnight at 4°C with antibodies against NF-κB (1:400), E2F1 (1:200), or CD70 (1:200). Secondary Alexa Fluor antibodies were applied for 45 min, followed by DAPI staining. Imaging was performed using a Zeiss Axioskop microscope.

For paraffin-embedded skin tissue, antigen retrieval was performed in sodium citrate buffer (10 mmol/L, pH 6.0). Sections were blocked and incubated overnight with CD70 (1:100, human samples) or with PCNA (1:600, mouse samples), MCP-3 (1:200, mouse samples), and IL-6 (1:200, mouse samples). After incubation with Invitrogen secondary antibodies (1:1000), autofluorescence was reduced using the TrueView Autofluorescence Quenching Kit (Vector Laboratories, Newark, CA).

**Apoptosis assay**

Apoptosis was measured using Annexin V-FITC/PI dual staining followed by flow cytometry. HaCaT cells were transduced with control or E2F1-targeted short hairpin RNA, harvested at 12 h and 24 h following SSL exposure or under untreated conditions (no SSL) as controls, and stained using the Annexin V-FITC Apoptosis Detection Kit (BD Biosciences) according to the manufacturer’s instructions. Briefly, cells were washed in cold PBS, resuspended in binding buffer, and incubated with Annexin V-FITC and PI for 15 minutes at room temperature in the dark. Stained cells were analyzed using a BD LSRFortessa flow cytometer, and data were processed using FlowJo software. Early (Annexin V + /PI -) and late (Annexin V + /PI +) apoptotic cells were quantified, and total apoptosis was calculated.

**Cell cycle analysis**

Cell cycle distribution was determined by PI staining and flow cytometry. Briefly, HaCaT cells were transduced with control or E2F1-targeted short hairpin RNA, then harvested at 12 h and 24 h following SSL exposure or under untreated conditions (no SSL) as controls. Cells were washed with PBS and fixed in 70% ethanol at -20 °C overnight. Cells were then incubated with RNase A (100 μg/ml) and stained with PI (50 μg/ml) in PBS for 30 minutes at room temperature in the dark. Flow cytometry was performed using a BD LSRFortessa (BD Biosciences), and the percentages of cells in G1, S, and G2/M phases were quantified using FlowJo software.

**Luciferase Assay**
 HaCaT or A431 cells (shCon or shCD70, 8 × 10⁴ cells per well) were seeded in 24-well plates and cultured for 24 h. Cells were transfected with NF-κB or AP-1 luciferase reporter plasmids along with a β-galactosidase plasmid as an internal control using iMFectin Poly DNA Transfection Reagent (GenDEPOT, Katy, TX) according to the manufacturer’s instructions.

For HaCaT cells, the medium was replaced with fresh medium, and cells were or were not exposed to solar-simulated light (SSL) at 60 kJ/m² UVA and 2.9 kJ/m² UVB. Cells were collected at 0 min, 5 min, 10 min, 30 min, 1 h, 3 h, and 24 h post-irradiation. For A431 cells, cells were treated or not treated with 20 ng/µL TPA (Sigma-Aldrich, St. Louis, MO) for 24 h following transfection. For promoter activity assays, A431 cells were transfected with either the empty pGL3 promoter vector or the CD70-pGL3 promoter vector, with or without E2F1 expression, or with wild-type versus mutant CD70-pGL3 promoter constructs (shCon or shE2F1).

Luciferase and β-galactosidase activities were measured using the Luminoskan Ascent and Multiskan MCC systems (Labsystems, Helsinki, Finland). Luciferase activity was normalized to β-galactosidase activity for all experiments to ensure accurate and reliable results.

**Human cytokine antibody array**

RayBio® Human Cytokine Antibody Arrays C5 (RayBiotech, Inc., Norcross, GA) analysis was performed following the manufacturer’s protocol using cell lysates pooled from multiple samples. Protein concentrations were determined using a Protein Assay Kit (Bio-Rad Laboratories, Hercules, CA), and 500 μg of protein per sample were used. Lysates were diluted in blocking buffer to a final concentration of 5 mg/mL. Array membranes were pre-incubated with blocking buffer for 30 min at room temperature, after which the buffer was removed. Each assay well was loaded with 1 mL of the diluted protein sample and incubated overnight at 4°C. Membranes were washed 3 times with 2 mL Wash Buffer I (5 min per wash, room temperature), followed by two washes with 2 mL Wash Buffer II under the same conditions. Biotin-conjugated antibodies (1 mL per well) were added, and membranes were incubated overnight at 4°C. After washing with Wash Buffers I and II, 2 mL of HRP-streptavidin solution were added, and membranes were protected from light with adhesive film and aluminum foil during a 2 h incubation at room temperature. Membranes were washed again with Wash Buffers I and II, followed by chemiluminescent detection. Signal intensities were quantified using ImageJ software, and data analysis was performed according to the manufacturer’s instructions.

**References**

1.	Martin M. Cutadapt removes adapter sequences from high-throughput sequencing reads. EMBnet journal **2011**;17:10-2

2.	Kim D, Paggi JM, Park C, Bennett C, Salzberg SL. Graph-based genome alignment and genotyping with HISAT2 and HISAT-genotype. Nature biotechnology **2019**;37:907-15

3.	Kim D, Langmead B, Salzberg SL. HISAT: a fast spliced aligner with low memory requirements. Nature methods **2015**;12:357-60

4.	Pertea M, Kim D, Pertea GM, Leek JT, Salzberg SL. Transcript-level expression analysis of RNA-seq experiments with HISAT, StringTie and Ballgown. Nature protocols **2016**;11:1650-67

5.	Kovaka S, Zimin AV, Pertea GM, Razaghi R, Salzberg SL, Pertea M. Transcriptome assembly from long-read RNA-seq alignments with StringTie2. Genome biology **2019**;20:1-13

6.	Pertea M, Pertea GM, Antonescu CM, Chang T-C, Mendell JT, Salzberg SL. StringTie enables improved reconstruction of a transcriptome from RNA-seq reads. Nature biotechnology **2015**;33:290-5

7.	Wang Q, Zhang T, Chang X, Lim DY, Wang K, Bai R*, et al.* ARC is a critical protector against inflammatory bowel disease (IBD) and IBD-associated colorectal tumorigenesis. Cancer research **2020**;80:4158-71
